# Distribution and protection of *Thesium chinense* Turcz. under climate and land use change

**DOI:** 10.1038/s41598-024-57125-8

**Published:** 2024-03-18

**Authors:** Boyan Zhang, Bingrui Chen, Xinyu Zhou, Hui Zou, Detai Duan, Xiyuan Zhang, Xinxin Zhang

**Affiliations:** Heilongjiang Genuine Wild Medicinal Materials Germplasm Resources Research Center, School of Life Sciences and Technology, Harbin Normal University, Harbin, 150025 China

**Keywords:** Climate change, Land use change, Species distribution model, *Thesium chinense*, Wild tending, Ecology, Ecology, Climate-change ecology, Ecological modelling

## Abstract

Wild medicinal plants are prominent in the field of Traditional Chinese Medicine (TCM), but their availability is being impacted by human activities and ecological degradation in China. To ensure sustainable use of these resources, it is crucial to scientifically plan areas for wild plant cultivation. *Thesium chinense*, a known plant antibiotic, has been overharvested in recent years, resulting in a sharp reduction in its wild resources. In this study, we employed three atmospheric circulation models and four socio-economic approaches (SSP1-2.6, SSP2-4.5, SSP3-7.0, and SSP5-8.5) to investigate the primary environmental factors influencing the distribution of *T. chinense*. We also examined changes in its suitable area using the Biomod2 package. Additionally, we utilized the PLUS model to project and analyze future land use changes in climate-stable regions for *T. chinense*. Our planning for wild tending areas of *T. chinense* was facilitated by the ZONATION software. Over the next century, the climate-stable regions for *T. chinense* in China is approximately 383.05 × 10^4^ km^2^, while the natural habitat in this region will progressively decline. Under the current climate conditions, about 65.06% of the habitats in the high suitable areas of *T. chinense* are not affected by future land use changes in China. Through hotspot analysis, we identified 17 hotspot cities as ideal areas for the wild tending of *T. chinense*, including 6 core hotspot cities, 6 sub-hotspot cities, and 5 fringe hotspot cities. These findings contribute to a comprehensive research framework for the cultivation planning of *T. chinense* and other medicinal plants.

## Introduction

Climate plays a crucial role in determining species distribution. The response of vegetation to climate change and the regional changes in plant distribution due to climate are important research areas^1,2^. Projections indicate that global warming will lead to an average surface temperature increase of 1.1 °C to 6.4 °C by the end of the twenty-first century, significantly impacting terrestrial ecosystems and increasing the risk of species extinction^3,4^. To adapt to these changing conditions, plants can modify their ecological niche and distribution areas to match the new environment^5,6^. However, climate change will bring uncertainties to the cultivation of medicinal plants^7^. Blind expansion of medicinal plants without scientific guidance not only jeopardizes their quality but also misallocates resources^8^. Although long-term field trials have traditionally been reliable for identifying suitable cultivation areas, they require extensive resources and observation of multiple growth cycles^9^. Thus, it is imperative to predict the potential suitable areas for medicinal plants under future climate change in order to better understand their cultivation potential in new habitats.

The loss of habitats due to changes in land use will worsen the impact of climate change on species and ecological dynamics globally^10^. Most studies that explore changes in plant species distribution and their future distribution only consider climate factors, with little attention paid to the synergistic effects of habitat and climate change^11–13^. However, in exploring changes in plant distribution, climate change-driven range expansion occurs in a spatial context. Each species has its own unique habitat requirements, meaning that while certain regions may be suitable for species survival, they may not serve as suitable habitats for natural populations due to land use limitations. With increasing human activity, it is expected that 10% to 20% of natural grasslands and forests will be replaced by agriculture and urban infrastructure by 2050, leading to substantial habitat loss for most wild species^14,15^. Therefore, it is necessary to explore the impact of land use change on species' habitat. Integrating both climate and land use changes is essential for gaining a more accurate understanding of species' distribution range^16,17^.

*Thesium chinense* Turcz. is a semi-parasitic perennial herb of the genus *Thesium* in the Santalaceae. Its wild population is distributed in China, Japan, Korea, and other places^18^. As an important medicinal plant in China, dried whole grass of *T. chinense* has significant anti-inflammatory and analgesic effects. It is often used to prevent and treat all kinds of acute inflammation, so it has the reputation of the plant antibiotic^19^. In recent years, due to the deterioration of the ecological environment caused by excessive harvesting and the intensification of human activities, the population of *T. chinense* has sharply decreased throughout China and even disappeared in some ecological areas, seriously reducing the production of *T. chinense*^20^. With the increasing demand from medicinal herb manufacturers, the supply–demand contradiction has become more prominent^21^. However, the current system of artificial breeding of *T. chinense* is not fully developed to meet the rapid growth of the *T. chinense* industry^20^. Wild tending involves boosting the population of a target species in its native or similar environment using natural or artificial methods that align with the species' growth characteristics and ecological requirements. This approach is crucial in enabling sufficient resource availability for human use while maintaining a balanced community^22,23^. Therefore, planning a wild tending area for *T. chinense* is very important.

Many studies have shown that under the influence of climate change and human activities, the suitable areas and habitat areas of most medicinal plants are gradually reduced, and population breeding is threatened^24–27^. *T. chinense* may also face this threat, consequently leading to shortages in medicinal resources. This study integrates the synergistic effects of climate change and land use change, and plans a wild tending area for *T. chinense* in China, aiming to provide more effective references for the protection and sustainable use of resources of *T. chinense* in China. The purpose of this study is to: (1) Determine the main environmental factors limiting the distribution of *T. chinense* by modeling the complete ecological niche of *T. chinense*. (2) Investigate the distributional shifts of *T. chinense* in response to climate change. (3) Obtain a climate stable area used suitable layers of *T. chinense* under different climate scenarios, and analyzed the changes in the habitat of *T. chinense* in this area. (4) Based on climate and land use change, develop strategies and outline management plans for establishing wild tending areas for *T. chinense* in China. (5) Offer a reference framework for the wild tending planning of other medicinal plants.

## Materials and methods

### Construction of species distribution model

#### Collection of occurrence data

**Figure 1:**
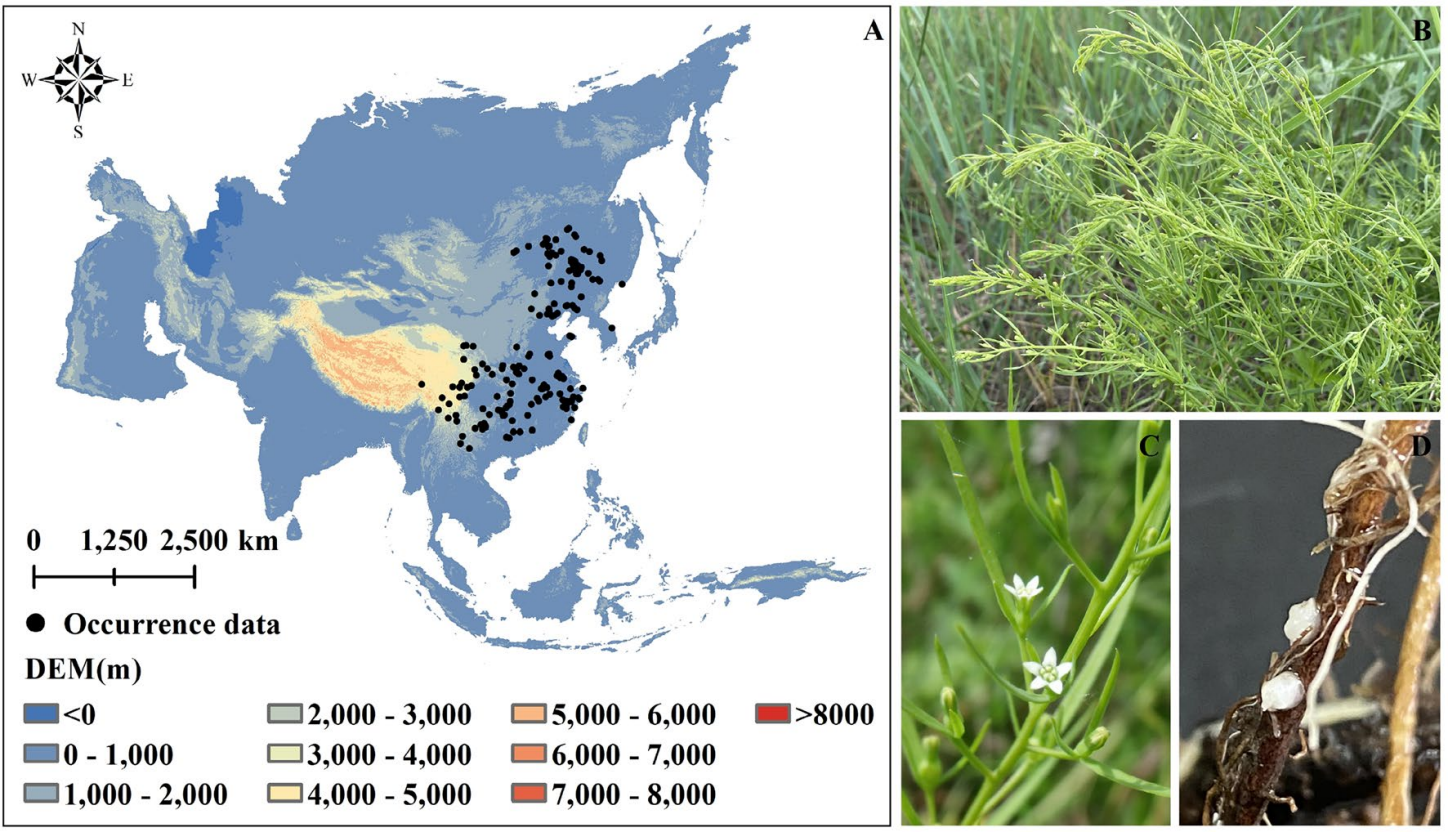
Morphological characteristics and occurrence data of *T. chinense*. (**A**) Occurrence data; (**B**) Plants of *T. chinense*; (**C**) Flowers of *T. chinense*; (**D**) Haustoriums of *T. chinense*. The map using ArcMap 10.5 sofware (URL: https://www.arcgis.com/index.html).

The geographical occurrence data of *T. chinense* were obtained by three methods: (1) Field investigation. An extensive survey was conducted from May 2018 to July 2022. (2) Network database. GBIF (https://www.gbif.org), CVH (http://www.cvh.ac.cn), and NSII (http://www.nsii.org.cn) and TSRSP (http://mnh.scu.edu.cn) have collected geographic location information with precise X and Y (latitude and longitude) and time range from 2000 to the 2022. (3) Literature search. Through the above methods, a total of 271 geographic occurrence data were obtained. Used Google earth (Google Earth USA) to reject occurrence data in construction land, cultivated land, and waters. Additionally, we mitigated spatial autocorrelation by removing duplicate occurrences within a 10 km using the "spThin" package^28^. Finally, 174 occurrence data were retained for modeling (Fig. 1).

#### Environmental factors

Using 22 environmental factors (including climate, soil and biology) to study the changes in the distribution of *T. chinense* (Table S1). In order to match the environmental factors with the current climate scenario, based on the monthly temperature and precipitation data provided by the Worldclimate database (v2.1), we calculated 19 climate data in the time range of 2000–2018 through the “biovar” package. Based on the biological characteristics of *T. chinense*^21^, we selected five soil factors (T_Clay、T_Gravel、T_Sand、T_OC and T_pH_H_2_O), which were downloaded from HWSD (Harmonized World Soil Database). As a semi-parasitic plant, vegetation (host) is the prerequisite for the growth of *T. chinense*. Therefore, we included NDVI factors that can reflect vegetation growth in the ensemble species modeling. NDVI data was downloaded in MODIS (https://www.earthdata.nasa.gov). We averaged the NDVI data from 2000 to 2020 according to the month of the growth period (May to July) of *T. chinense*^18^, and finally obtained layer was required for the species distribution model modeling.

High correlation between environmental factors can result in overfitting of the species distribution model. To mitigate this, we initially constructed the model using MAXENT 3.4.4 software without parameter adjustments, performing 10 repeated modeling. Next, we eliminated environmental factors that contributed less than 1%. We then utilized the ENMTools software, following the approach outlined by Warren et al. (2010)^29^, to analyze the remaining environmental factors' correlation. We screened out two environmental factors with an absolute correlation coefficient (|r|) ≥ 0.7 and removed the one with a relatively lower contribution rate. Afterwards, we calculated the variance inflation factor (VIF) of the remaining environmental factors and removed environmental factors with |VIF|≥ 5. After these steps, we retained a total of 9 environmental factors for the final modeling.

To project the distribution changes of *T. chinense* in different periods (2050s: 2041–2060, 2070s: 2061–2080, 2090s: 2081–2100) in the future, we selected three widely used atmospheric circulation models (MIROC-ES2L, CNRM-CM6-1, and MRI-ESM2-0) to build species distribution model in the future. Each atmospheric circulation model includes four shared socio-economic pathways: SSP1-2.6, SSP2-4.5, SSP3-7.0, and SSP5-8.5, with 12 climate scenario combinations. Among four selected shared socio-economic pathways, the low to high radiative forcing scenarios range from SSP1-2.6 to SSP5-8.5^30^. The data from the three climate models within the same carbon emission scenario and year were averaged using ArcMap10.5 software. The environmental data had a spatial resolution of 2.5 min.

#### Construction of species distribution model

Ten modeling algorithms (GBM, CTA, GLM, MARS, RF, GAM, ANN, FDA, SRE, and MAXENT) provided by "Biomod2" package were used to predict the potential distribution of *T. chinense*. All models use default parameters except the MAXENT model. Accuracy of the MAXENT is influenced by parameter settings. We tested the complexity and performance under different settings of eigenvalues (FC) and multiplicators (RM) used the kuenm package in R 3.6.3^31^. Candidate models were created by combining 17 RM values and all 31 possible combinations of five FC (L, Q, H, P, T). The optimal model was determined based on the Akaike Information Criterion (AICc, deltaAICc = 0)^31^. The optimized parameters were FC = LQPT and RM = 3.

In species distribution modeling, 70% of the occurrence data was selected as training data, while the remaining 30% was used as testing data. This process was repeated five times. To reduce spatial bias and better simulate the actual distribution of species, we created 5,000 pseudo-absence points, repeated 3 times and modeled. In the end, 150 layers were generated.

Each model was evaluated using the TSS^32,33^. The closer the TSS value are to 1, the more reliable the prediction will be^34,35^. The final species distribution layer was calculated based on the model with average TSS (≥ 0.8) values.

#### Division of suitable areas and climatic characteristics

When converting continuous predictions into a Boolean classification of "suitable" and "unsuitable", it is crucial to choose an appropriate threshold value^36^. We used the maximum training sensitivity plus specificity threshold, which maximized the True Skill Score (TSS) and generated binary maps^37,38^. The selected threshold value (P) for the suitable areas of *T. chinense* was 0.374. Moreover, these areas were categorized into three levels: low suitable areas (0.374 ≤ P < 0.5), medium suitable areas (0.5 ≤ P < 0.75), and high suitable areas (P ≥ 0.75). We used SDMtools to compare the spatial changes of the suitable areas of *T. chinense*.

To analyze the change of environmental characteristics within the suitable areas of *T. chinense*, we randomly selected 5000 points from the current suitable area. From these points, we extracted values from the dominant environmental factors of different climate layers. R 4.2.0 software was used to calculate the 95% quantile and average value of the extracted values, providing insight into the potential environmental pressures that *T. chinense* may face in the future^39^.

#### Analysis of multivariate environmental similarity surface (MESS)

The Do MESS analysis when projecting function analysis in MAXENT 3.4.4 was used to calculate the multiple environmental similarity surface (MESS) of the complete ecological niche of *T. chinense*, and to analyze the similarity (S) between the future climate conditions and the current climate. When S > 0, it indicates that the climate at that point has not been significantly affected. When S < 0, it indicates that the climate at that point has been severely affected, indicating that the value of one or more bioclimatic variable have exceeded the range of the reference value. A smaller S means that the climate factors in the region are less similar.

### Future land use simulation

#### Land use and driver data

To simulate the future land use in China, we downloaded land use data from RESDC (Resource and Environment Science and Data Center), including 2010 and 2015. These data were classified according to the classification system of China's National Land Use and Cover Change (CNLUCC) (Table S2) using ArcMap10.5 software. The reclassified data included waters, cultivated land, grassland, construction land, forest, and unused land. Grassland was defined as a type of land cover that is dominated by herbaceous plants and has a coverage exceeding 5%. It encompasses grazing-dominated shrub grasslands as well as sparse forest grasslands with a canopy density below 10%. The classified grassland region corresponds to the habitat previously recorded by *T. chinense*, and thus, we considered it to be the natural habitat of *T. chinense*. The driving factors were divided into physical geography, human disturbance, and bioclimate (Fig. S1). Physical geographic factors included elevation (WorldClim Database v2.1), soil erosion (RESDC), and distance from water. Distance from the water was calculated based on water system data (RESDC) using the EuclideanDistance tool in ArcMap10.5. Human disturbance factors included the population data and GDP data of 2015 downloaded from the RESDC, and the distance from road and railway (DRA) calculated by EuclideanDistance tool based on the road data and railway data of RESDC. Bioclimatic factors included annual precipitation and mean temperature data from WorldClim Database v2.1 in 2015. Use the resampling tool in ArcMap10.5 to unify all of the above data layer resolutions to 2.5 min.

#### Model building

The PLUS model is a novel land use simulation model based on cellular automata, which offers distinct advantages in investigating the drivers of land use change and dynamically simulating changes in various land use types, particularly for forest and grassland patches^40,41^. To simulate future land use, the model extracts samples representing the mutual transformation of different land use types from time-period data for training purposes. The transformation probability is then used to simulate future land use. By employing the random forest algorithm, the model calculates the expansion and driving factors of different land use types (Tables S3 and S4), revealing the development probability and the contribution of driving factors to their expansion during a specified period^40,42^. The PLUS Model comprises two main components: LEAS (Land Expansion Analysis Strategy) and CARS (Cellular Automata Model Based on Multitype Random Patch Seeds). Detailed formulas for module calculations can be found in relevant literature^43–45^.

### Planning of wild tending area

To serve the wild tending action of *T. chinense* under climate change and land use change, the grassland types in 2015, 2050, 2070, and 2090 were superimposed to obtain grass stability region this century. Considering the prohibition of the development of nature reserves, the existing protection areas were excluded from the current high suitable areas. By using the core area zonation algorithm in ZONATION4.0 software (https://www.helsinki.fi/), the layers of the high suitable areas in different climate scenarios in the grass stability region were superimposed. Each layer was assigned a weight of 1, while other parameters were set to default values^46,47^. The natural discontinuity method was used to classify the output layers from 0 to 1, and the wild tending areas with low, medium, and high suitability of *T. chinense* were obtained.

## Result

### Accuracy of ensemble species distribution model

**Figure 2:**
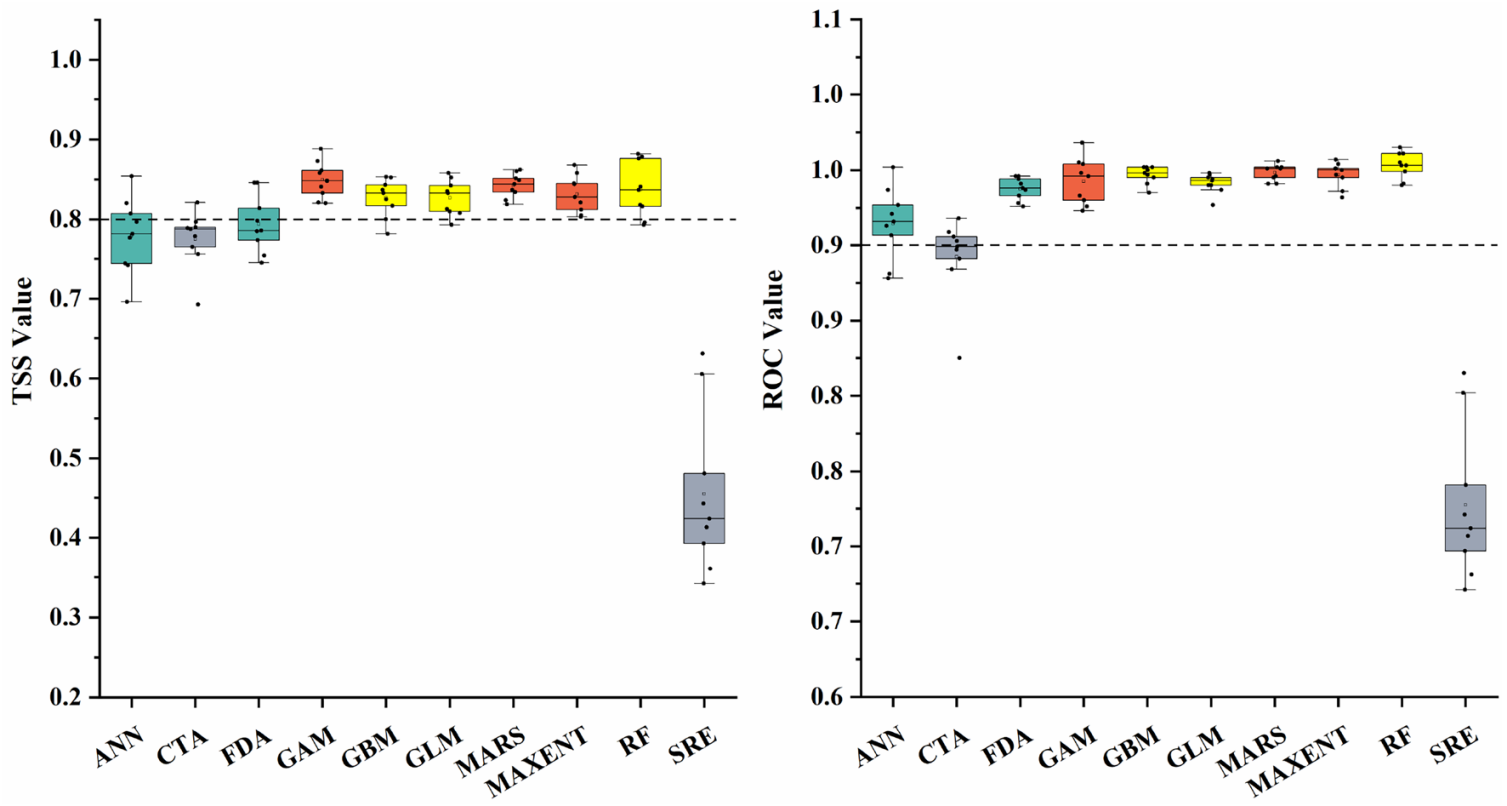
Prediction accuracy evaluation of different models.

The results of ensemble species distribution model showed that there were differences in the prediction accuracy of 10 models, among which RF, GBM, MARS, MAXENT, GLM, CTA, FDA and GAM met the standard of ensemble model establishment (Fig. 2). All replicates of the SRE model failed to meet the specified TSS and ROC value standards, and only a few replicates of CTA reached the standard. Therefore, these two models were not involved in ensemble models.

### Relationship between environmental factors and distribution of *T. chinense*

**Figure 3:**
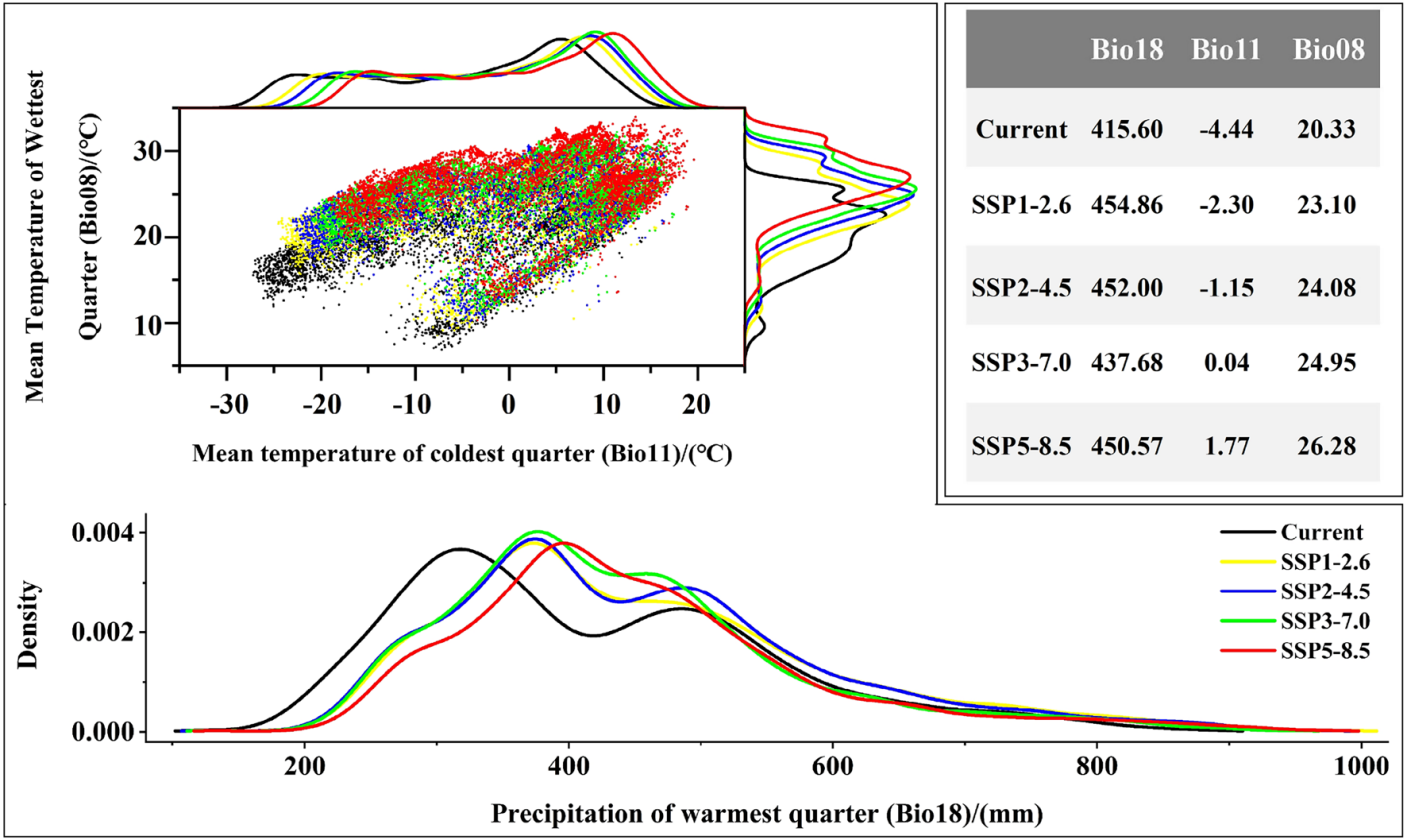
Changes of the dominant environmental factors in the current suitable area of *T. chinense* in the 2090s.

After screening, the 9 environmental factors were used to establish the model and their importance were shown in Table S5. Among them, Bio18 is the most important environmental factor, followed by Bio11 and Bio08. The suitable range of main environmental factors in the current suitable area of *T. chinense* was shown in Table S6. Specifically, the suitable range of Bio18 is 216.27 mm ~ 741.47 mm. The suitable value distribution of Bio11 was -25.03 °C to 11.62 °C. The lowest suitable value for Bio08 was 9.80 °C, and the highest value was 26.33 °C. In future, the value ranges of Bio18, Bio11, and Bio08 show an increasing trend in the current suitable area. As carbon emission concentrations continue to rise, the increase rate will escalate over time (Table S6). It is expected that under the climate scenario combination of 2090s-SSP5-8.5, Bio11 and Bio08 will rise to the highest level, with an increase of 6.21 °C and 5.95 °C compared with current. The highest value of Bio18 appears in the 2090s-SSP1-2.6 climate scenario combination, increasing by approximately 39.26 mm (Fig. 3).

### Suitable areas of *T. chinense* in current

**Figure 4:**
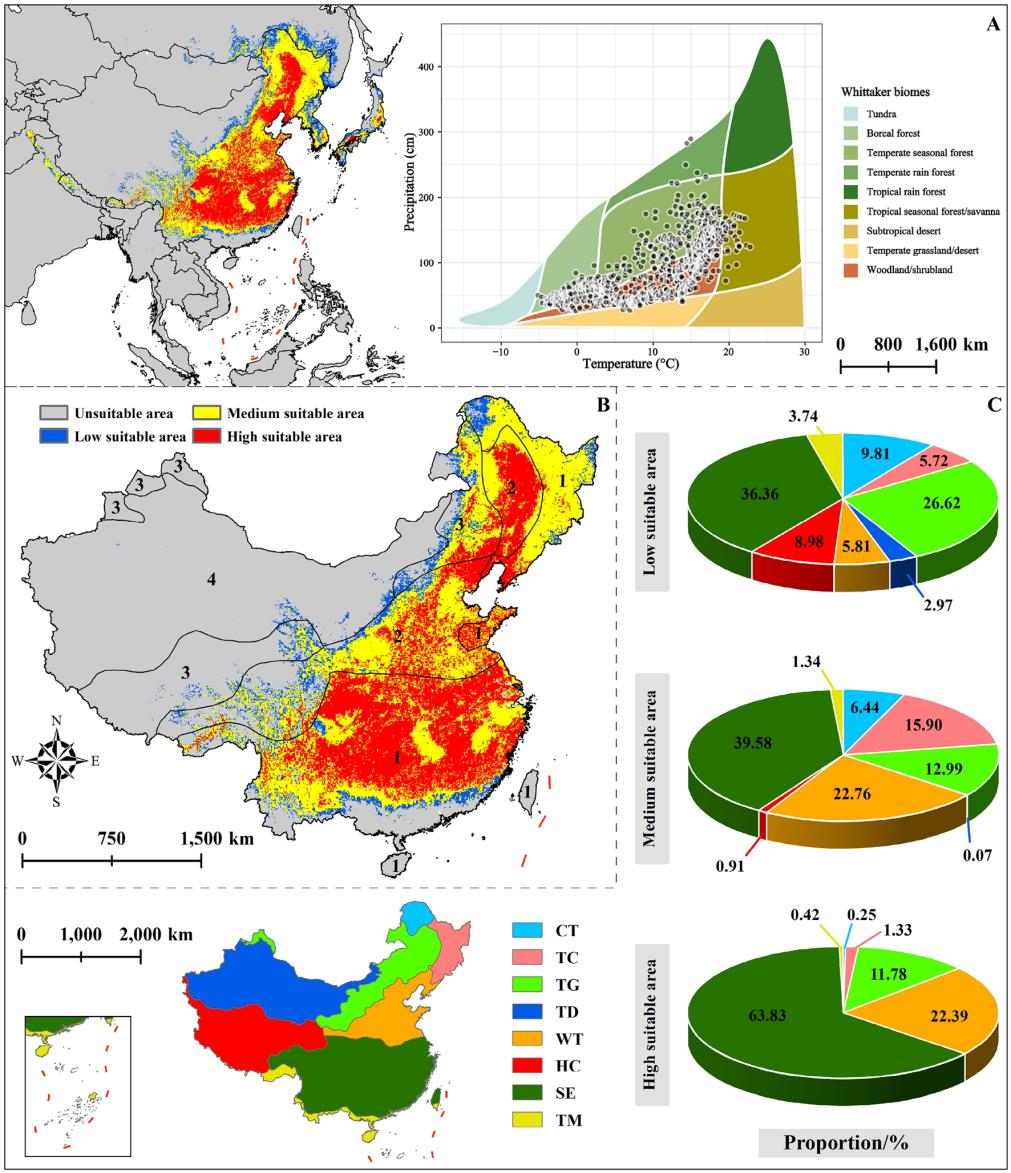
Distribution of *T. chinense*’s suitable areas. (**A**) Current distribution range ; (**B**) The distribution range and dry and wet areas of *T. chinense* in China, among which 1–4 represent: 1: humid area, 2: sub-humid area, 3: semi-arid area, 4: arid area; (**C**) The proportion of suitable areas of different grades of *T. chinense* in each vegetation areas in China, where the abbreviations represent *CT* Cold temperate coniferous forest region, *TC* Temperate coniferous and deciduous broad-leaved mixed forest region, *TG* Temperate grassland region, *TD* Temperate desert region, *WT* Warm temperate deciduous broad-leaved forest region, *HC* High cold vegetation region on the Qinghai Tibet Plateau, *SE* Subtropical evergreen broad-leaved forest region, *TM* Tropical monsoon forests, rainforest region. The map using ArcMap 10.5 sofware (URL: https://www.arcgis.com/index.html).

Under current (2000–2018) climate conditions, the complete ecological niche of *T. chinense* is primarily in three vegetation areas (Boreal forest, Temperate seasonal forest, and Shrubland) in the eastern of Asia (Fig. 4A). The total suitable areas approximately 552.90 × 10^4^ km^2^, with high, medium, and low suitability regions encompassing about 194.19 × 10^4^ km^2^, 259.33 × 10^4^ km^2^, 99.38 × 10^4^ km^2^, respectively. In China, the suitable areas of *T. chinense* occupy approximately 460.73 × 10^4^ km^2^, mainly distributed within humid and semi-humid regions, with a minor presence in the semi-arid of Northeast China (Fig. 4B).

The high suitable areas of *T. chinense* in China is approximately 189.47 × 10^4^ km^2^, as shown in Fig. 4C, mainly distributed in SE, WT and TG, accounting for 98.00% of total high suitable areas. There are also minor high suitable areas found in TC, TM and CT. Medium suitable areas surround the high suitable areas and range from the southern SE to the northern CT, covering an area of roughly 220.70 × 10^4^ km^2^. Low suitable areas is about 50.55 × 10^4^ km^2^, mainly distributed within SE and TG, accounting for about 36.36% and 26.62%, respectively (Fig. 4C).

### Changes in the suitable areas of *T. chinense* under future climatic conditions

The MESS under future climate shows that the climate in the majority of the study area shows relatively stable climate patterns in the future. However, climate anomalies are mainly distributed in Indonesia and Pakistan (Fig. S2). The area within the research area experiencing future climate anomalies (S < 0) gradually increases with years and carbon emissions. It reaches its maximum extent (328.16 × 10^4^ km^2^) under the climate scenario of 2090s-SSP5-8.5. The average S of the current 174 distribution points of *T. chinense* gradually decreases with years and carbon emissions. It reaches its minimum value (10.12) under the climate scenario of 2090s-SSP5-8.5.

**Figure 5:**
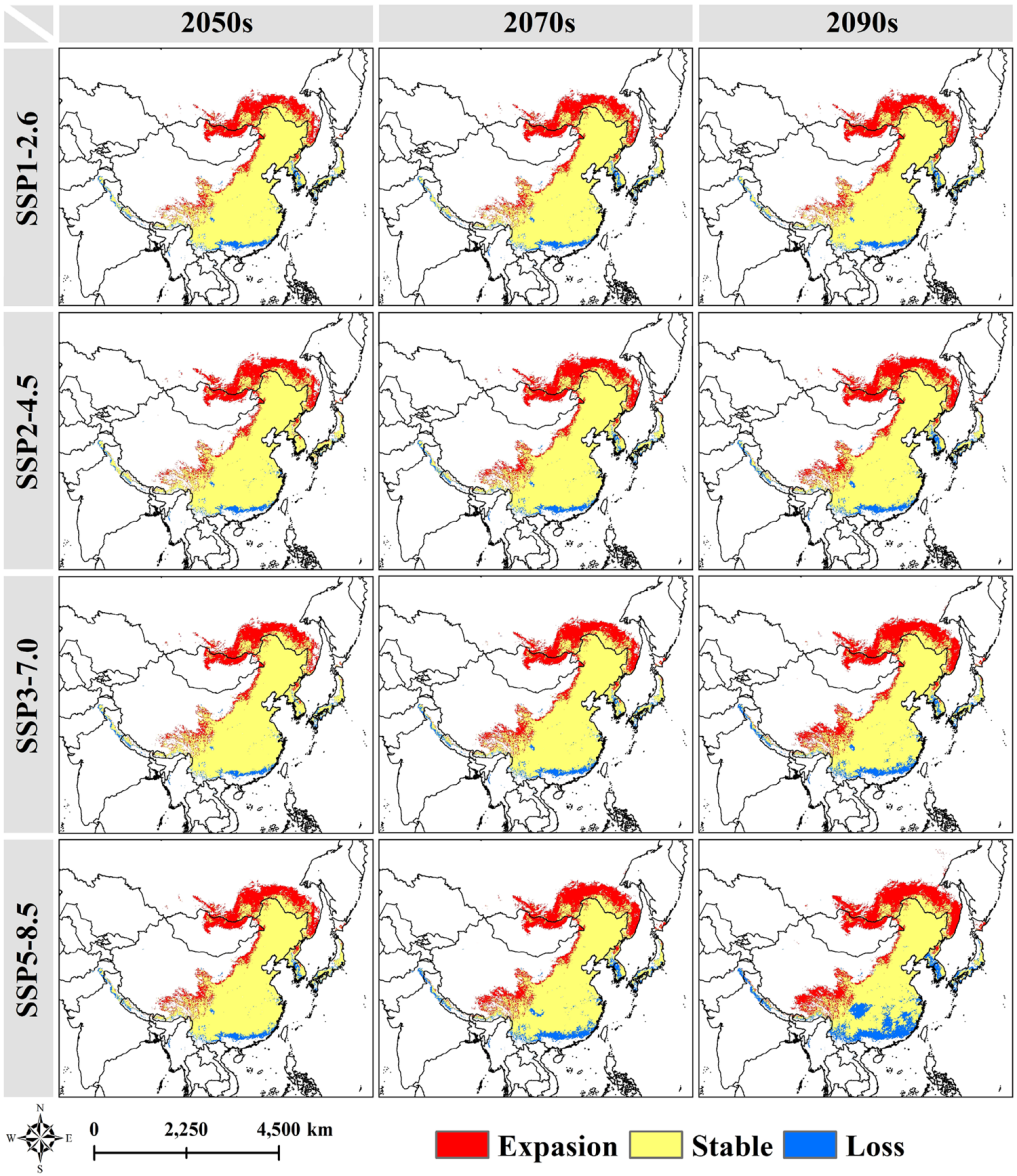
Spatial pattern variations of *T. chinense*.

**Figure 6:**
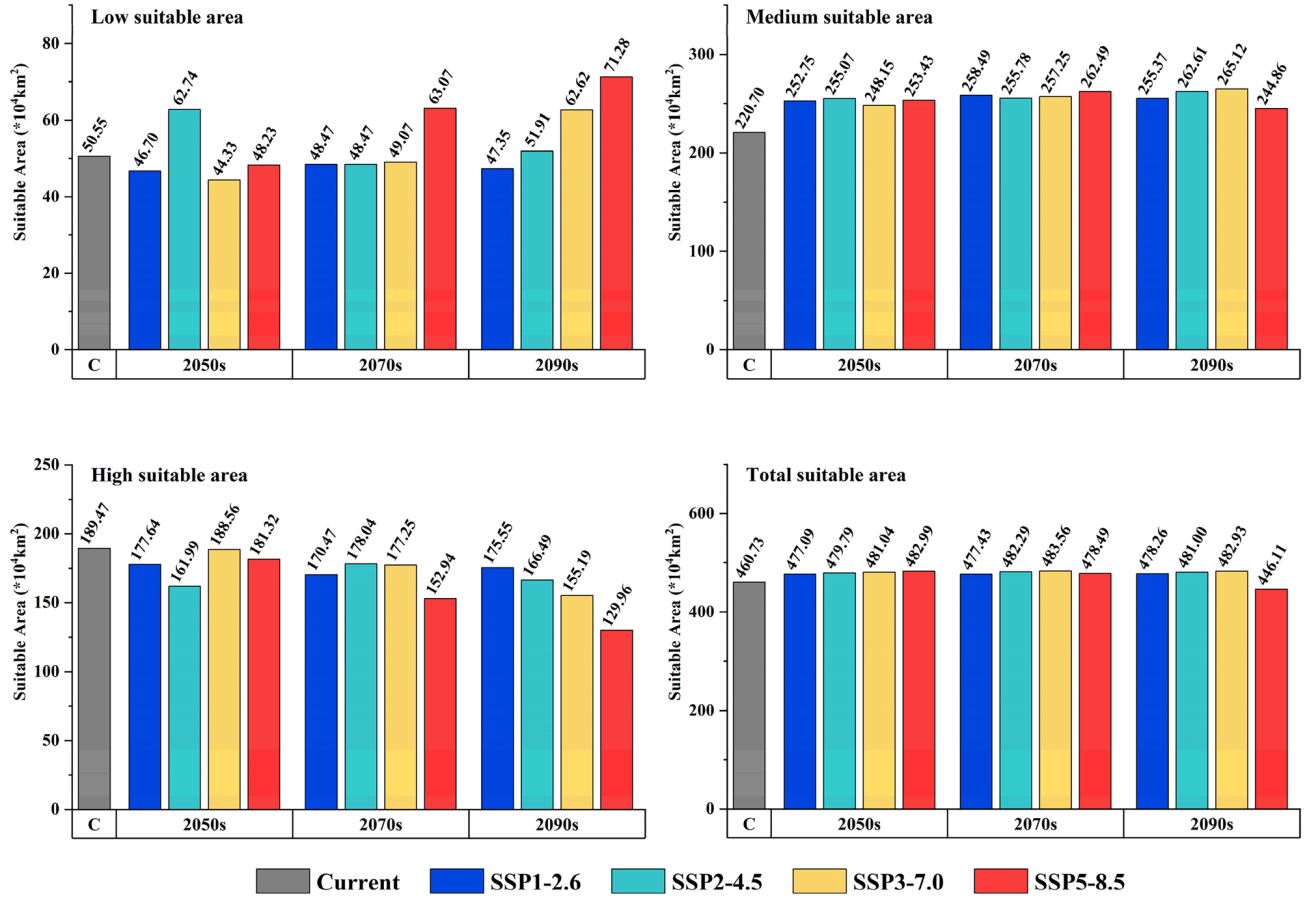
Changes of suitable areas of *T. chinense*.

Figures S3 and S4 showed the spatial distribution pattern and area change of suitable areas at each level of the complete ecological niche of *T. chinense*. In China, the suitable areas will still be mainly distributed in the eastern and southern of China under future climatic conditions. With the increase of carbon emissions and years, the suitable area of *T. chinense* will gradually move to the area with higher altitude and latitude, showing a significant expansion trend in the northern part of the current suitable area and Tibet, the reduction area is mainly distributed in the southern part of the suitable area (Fig. 5 and Fig. S5). Except for the climate scenario of 2090s-SSP5-8.5, the total suitable area of *T. chinense* in China will increase compared to the current situation in the future, and the total area of *T. chinense* will expand to the maximum (483.56 × 10^4^ km^2^) under the climate scenario of 2070s-SSP3-7.0 (Fig. 6). In all levels of suitable areas, high suitable areas show a decreasing trend, whereas the low suitable areas and medium suitable areas show an increasing trend. Specifically, in the 2090s-SSP5-8.5 scenario, high suitable areas experience the most significant reduction, shrinking by 31.41% compared to the present conditions In comparison, the low suitable areas are observed to expand to their maximum extent covering approximately 71.28 × 10^4^ km^2^ under the same scenario.

### Distribution and change of land use in climate-stable areas

**Figure 7:**
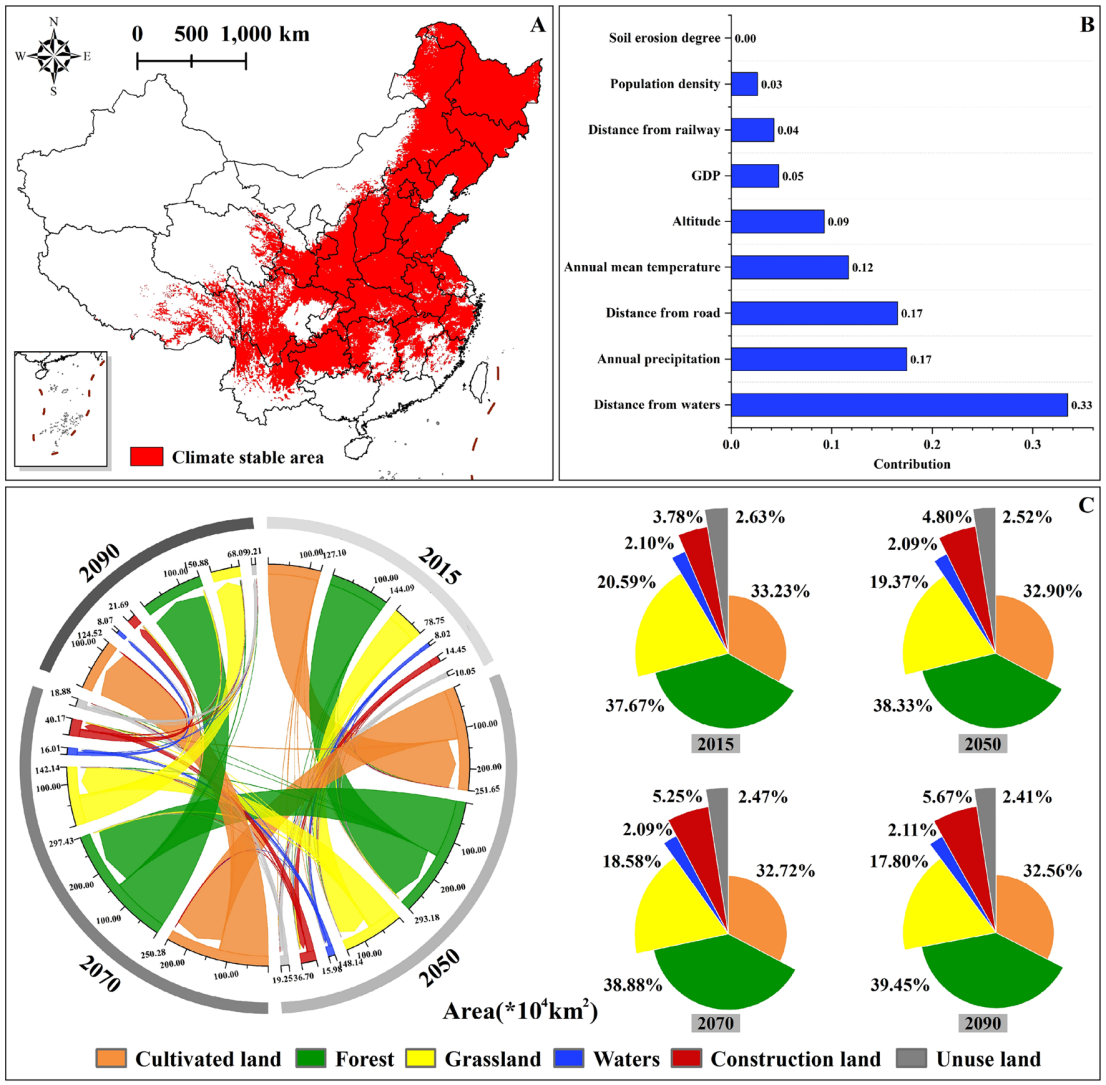
Map of the climate-stable areas and land use change. (**A**) Climate-stable areas of *T. chinense* under different climate scenarios in China; (**B**) Contribution of driving factors to the change of grassland; (**C**) Conversion and change of land use type area in 2015, 2050, 2070, and 2090 within the climate-stable areas of China. The map using ArcMap 10.5 sofware (URL: https://www.arcgis.com/index.html).

Kappa coefficient was used to verify the simulation accuracy between the simulation results in 2015 and the actual land use data in 2015. The results showed that the overall accuracy of the simulation results of land use change in 2015 was 0.992, with a Kappa coefficient of 0.990. This suggests a high level of reliability in the PLUS model, indicating its capability to forecast future land use distribution. The contribution degree of driving factors of each land use type change was shown in Table S7. Notably, the primary driving factor influencing the alteration of grassland areas (natural habitat of *T. chinense*) was the distance from water, followed by annual precipitation (Fig. 7B).

Under different climate scenarios, the total area of climate-stable areas of *T. chinense* in China is about 383.05 × 10^4^ km^2^ (Fig. 7A). According to the expansion and contraction trends of land use in 2010 and 2015, the distribution pattern of land use in 2050, 2070 and 2090 within the climate-stable areas is predicted (Fig. S6). Compared with 2015, the cultivated land, grassland and unused land within the climate stable area showed a downward trend, and decreased by 2.04%, 13.54% and 8.39% respectively by 2090 (Fig. 7C). In the future, the land use types transferred to grassland types mainly are forest, construction land and unused land. From 2050 to 2070, the areas will transfer from forest, construction land and unused land to grassland is about 1.25 × 10^4^ km^2^, 0.55 × 10^4^ km^2^ and 0.49 × 10^4^ km^2^. With the increase of the years, the transferred areas gradually increase. From 2070 to 2090, the area transferred from forest, construction land and unused land to grassland was about 2.27 × 10^4^ km^2^, 0.84 × 10^4^ km^2^ and 0.59 × 10^4^ km^2^. In future, grassland will mainly transfer to forest and construction land, with the transfer areas of 3.38 × 10^4^ km^2^ and 1.58 × 10^4^ km^2^ from 2050 to 2070, and 4.45 × 10^4^ km^2^ and 1.79 × 10^4^ km^2^ from 2070 to 2090 (Fig. 7C and Table S8). In the future, the area of construction land and forest waters in this region will show an increasing trend, especially the construction land. By 2090, the area of forest and water will increase by 4.72% and 0.72%, respectively. Construction land will increase to 21.69 × 10^4^ km^2^, expanded by about 50.07%.

### Wild tending areas of *T. chinense*

**Figure 8:**
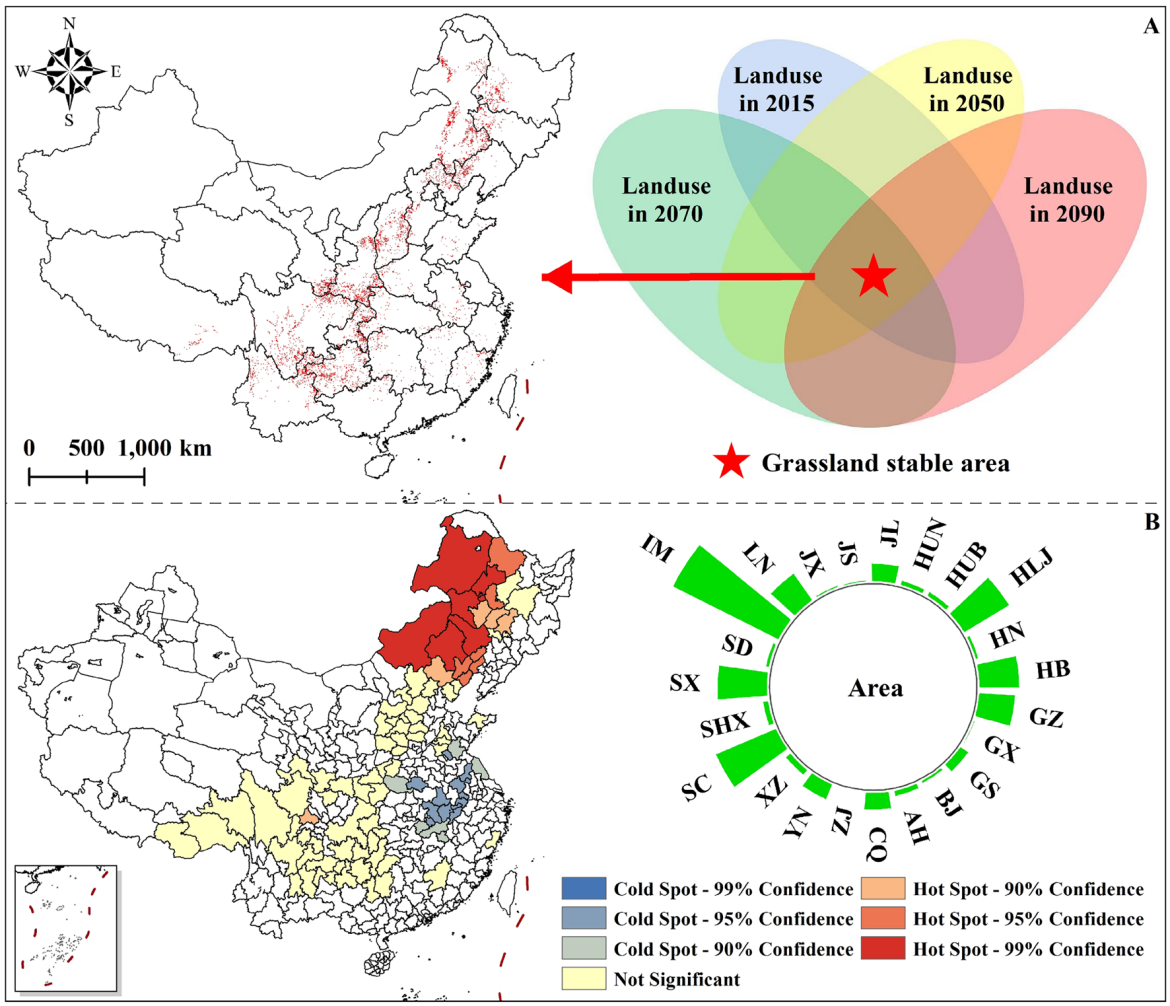
The distribution of grassland-stable areas and wild tending areas in the current high suitable areas. (**A**) The grassland-stable areas within the potential high suitable areas of *T. chinense* under the current climate scenario in China; (**B**) Analysis on the provincial area and hot spots at city level of high suitable wild tending area, where the abbreviations represent *IM* Inner Mongolia Autonomous Region, *SC* Sichuan Province, *HLJ* Heilongjiang Province, *SX* Shanxi Province, *HB* Hebei Province, *GZ* Guizhou Province, *LN* Liaoning Province, *JL* Jilin Province, *CQ* Chongqing City, *YN* Yunnan Province, *GS* Gansu Province, *XZ* Xizang Autonomous Region, *AH* Anhui Province, *SHX* Shaanxi Province, *HUB* Hubei province, *HUN* Hunan Province, *SD* Shandong Province, *HN* Henan Province, *BJ* Beijing, *JX* Jiangxi Province, *GX* Guangxi Zhuang Autonomous Region, *JS* Jiangsu Province, *ZJ* Zhejiang Province. The map using ArcMap 10.5 sofware (URL: https://www.arcgis.com/index.html).

Under the current climate situation, approximately 65.06% of habitats in high suitable areas are projected to remain unaffected by future land use changes in China. These areas are expected to serve as stable habitats for *T. chinense* over the next century (Fig. 8A). High, medium, and low suitable wild tending areas were obtained by superposing the suitable layers of *T. chinense* under different climate scenarios in the region. IM has the largest area of high suitable wild tending area, followed by SC, HLJ and SX (Fig. 8B). Based on the hot spot analysis of the area of the high suitable wild tending areas (administrative units), the results showed that there are 17 hotspot cities that are most suitable for planning the wild tending areas of *T. chinense.* Among them, there are 6 core hotspot cities: Hulunbuir City, Qiqihar City, Xing'an League, Xilingol League, Chifeng City and Tongliao City; 6 sub-hotspot cities: Heihe City, Daqing City, Fuxin City, Chaoyang City, Jinzhou City and Huludao City; and 5 fringe hotspot cities: Baicheng City, Songyuan City, Changchun City, Chengde City and Chengdu City。(Fig. 8B, Fig. S7).

## Discussion

### The possible effects of environmental factors on the population of *T. chinense*

Various environmental factors play a role in influencing the distribution of *T. chinense*, although only a subset significantly influences its habitat. Among these factors, water-related environmental factors (Bio18) have the greatest influence on the distribution of *T. chinense* under current climate conditions, which aligns with previous studies^3^. *T. chinense* is commonly found in shaded or humid areas and is not tolerant to drought. Insufficient rainfall inhibits its growth and development^5,18^. In the summer, excessive moisture in the soil or water on the surface can lead to root rot and hinder its growth^5,48^. Temperature also significantly influences the distribution of *T. chinense*, with Bio11 and Bio08 playing important roles. Bio11 exhibits a wide suitable temperature range (− 26.47 °C to 13.22 °C), indicating the species' high cold resistance. During harsh winter conditions, *T. chinense* undergoes overwintering and dormancy processes, during which starch degradation into soluble sugars primarily occurs via starch phosphorylation. This ensures the availability of sufficient carbohydrates to sustain growth and enhance stress resistance in low-temperature environments, facilitating smooth overwintering^49^. Additionally, under low-temperature stress, soluble sugars form small molecular solutes, further augmenting the species' ability to withstand severe cold^50^. On the other hand, Bio08 represents the mean temperature of wettest quarter. The spatial and temporal distribution of precipitation in Asia is extremely uneven. Precipitation is more abundant during summer, with the wettest quarter primarily concentrated in this season^51^. When the summer temperature is greater than 30 °C, seedling collapse occurs in the *T. chinense*. And the mortality rate of annual individual seedling collapse is relatively high, which is basically unable to survive^49^. This seriously affects the renewal of the *T. chinense* population and the continuation of its species. The range of Bio08 (9.08 °C ~ 26.38 °C) well reflects this phenomenon^52^.

By the 2090s, the Bio11 and Bio08 within the suitable area of *T. chinense* have risen by 6.32 °C and 6.42 °C, respectively. For high-latitude areas within the suitable area, the increased winter temperature may prevent *T. chinense* from entering its hibernation period, leading to continuous nutrient consumption and potentially adverse effects on the growth and reproductive capacity of the following year. For low-latitude areas in the suitable area, high temperatures may not only exacerbate the lodging of *T. chinense*, but also accelerate leaf senescence, reduce the reuptake rate of N and P, and weaken its ability to adapt to the environment^53,54^. Therefore, in a century of climate change, temperature increasing may be detrimental to the growth of *T. chinense*, reducing its ability to adapt to the environment and further threatening the population. By the end of this century, the Bio18 may increase to 438.51 mm. It is speculated that, with increasing summer precipitation, the negative effect of the single temperature factor (the increase in soil evaporation) on *T. chinense* may be counterbalanced by the increase in precipitation (the increase in soil moisture) to reach an equilibrium state. However, supposing there is further increase in precipitation or temperature, the balance may be disrupted, creating water or temperature stress. In that case, it may limit the growth of *T. chinense*.

### Distribution change of the population of *T. chinense*

Biomod2 was utilized to predict the distribution of *T. chinense* in Asia, and the results were not completely consistent with the regional niche results predicted by Tang et al. and P. Gao et al.^3,5^ using the MAXENT model. It is speculated that there are four reasons for this result.

The first point is the choice of modeling scope. In the existing studies, the methods of constructing species distribution models are divided into complete and regional niche models. Complete niche models, which can calibrate the presence and encompass a broader range of occupied environmental conditions, are considered more reliable for describing a species' climatic niche^55^. However, incorporating environmentally suitable but unoccupied areas during model training can diminish the capacity to predict the species' potential distribution^56^. Barve et al.^57^ advocate incorporating a geographical background that includes the species' historical locations for model training, validation, and comparison. In this study, *T. chinense*’s distribution was reviewed, and the region with the highest accessibility (Asia) was selected as the study area. Secondly, the use of ensemble species distribution model can reduce the instability of a single model^58,59^. Third, there is a serious bias in the occurrence data used by Tang and Gao et al. in constructing the model^3,5^. Our field investigation found *T. chinense* also distribute in the high latitude areas in the northern of China, contrasting with the limited or absent occurrence data in these regions in the mentioned studies. These data are predominantly concentrated in low latitude in the southern of China, which cannot objectively identify the niche of species^60^. In addition, we speculated that the environmental factors also contribute to the different results. As a semi-parasitic plant, vegetation (host) is the premise for the good growth of *T. chinense*, so biological factors play a important role in the distribution modeling of the species. At present, the inclusion of interspecific interactions is considered to be the main challenge for species distribution modeling^61^. Considering that there are many host species of *T. chinense*, mainly Asteraceae, Fabaceae, and Poaceae, which are widely distributed^62^, we included NDVI data reflecting vegetation growth in the species distribution model. Although NDVI data may not precisely describe specific vegetation conditions, we believe that the its inclusion may contributes to a more realistic representation of the niche.

Under the combination of future climate scenarios, the overall spatial distribution area of *T. chinense* will gradually expand to the high latitude and high altitude areas in the northwest of the current suitable area. This trend aligns with the observed changes in the spatial distribution of numerous species modeled at national or intercontinental scales^24,36,63–66^. Many *Thesium* species in low-latitude regions are facing a reduction in suitable habitats compared to other plant species within the genus. The results of the distribution models of *Thesium* species from the Cape Floristic Region indicate that over 50% of the species show a reduction trend, supported by our results^67^. Unfortunately, there are fewer distribution modeling studies on the complete ecological niche of *Thesium* species. Consequently, the actual situation may be even more dire than the aforementioned projections.

Future modeling results indicate that *T. chinense*'s suitable areas in China will predominantly expand into Tibet. Presently this area is in the semi-arid area, with sufficient soil moisture to meet the plant's growth requirements. However, due to the high altitude, the low temperatures make it unsuitable for the growth. Anticipated changes in the future involve the expansion of the plateau temperate zone in southern China northward, gradually replacing the plateau sub-cold zone in western China. This shift is expected to increase temperatures in the region^68^. Consequently, the climate conditions in this area may resemble those in the current suitable areas of *T. chinense*, potentially contributing to the expansion.

In the future, the disappearing areas of suitable areas of *T. chinense* are mainly located in the southern of China. As the climate warms, the region will change from the subtropical zone to the tropical zone^68^. This unfavorable shift in conditions is detrimental to the absorption of soil fertility by plant roots^69,70^, rendering the area unsuitable for *T. chinense* growth.

### Conversion characteristics of land use in the suitable area of *T. chinense*

Human activities are a major factor leading to global change, which overwhelm the natural changes brought about by climate change in the past few thousand years^71^. Human activities such as agriculture, forestry, and other land management have changed the entire landscape, thus affecting the flora and fauna communities of many ecosystems worldwide^72^. In China, the climate-stable areas of *T. chinense* are mainly distributed in humid and semi-humid areas, and the major changes of land use types in this area in the future are mainly construction land and grassland. It is predicted that the construction land in the region will expand rapidly in the next hundred years, an increase of about 46.94% compared with 2015. The distribution of construction land is mainly related to population density. Future population projections for China indicate a general pattern of dense population distribution in the southeast and sparse distribution in the northwest. Urban clusters are experiencing a gradual increase in both population size and concentration, leading to a more pronounced population clustering trend^73^. Therefore, our study predicted that the rapid growth of the construction land of *T. chinense* in the future climate-stable areas in China is in line with the actual situation.

Grassland ecosystem is the largest ecosystem in land and occupies an extremely important role in the terrestrial ecosystem. The impact of human activities on the grassland ecological system in recent years is gradually growing^74^. Since the twenty-first century, Chinese government has invested vigorously in ecological restoration projects and carried out ecological restoration work, such as returning farmland to grassland, which significantly affects the restoration of grassland soil nutrients (carbon)^75^. In the climate stable area suitable for the growth of *T. chinense* in the future, a small amount of cultivated land was transferred to grassland from 2015 to 2050, but there was no such transfer from 2050 to 2090. Therefore, the effect of returning farmland to grassland on soil nutrient recovery in the climate stable area of *T. chinense* is very small in the future. And in the future, the areas of construction land transferred to grassland in the region will increase yearly. It is estimated that by 2090, the areas of construction land transferred from 2070 will account for about 1.22% of the grassland areas in 2090. Large scale construction projects have not only seriously damaged grassland vegetation and topsoil, resulting in a sharp decline in grassland carbon storage that will take many years to recover, but they have also intensified climate change, thereby indirectly impacting the grassland ecosystem and causing certain adverse effects^76^. Therefore, in the context of land use change, the natural habitat suitable for the growth of *T. chinense* will face a huge threat.

### Planning for wild tending area of *T. chinense*

Under land use and climate change, although climate change increases the total suitable areas of *T. chinense*, it will seriously threaten the high suitable areas. Under different climate scenarios within stable climates conducive to *T. chinense* growth, land use changes lead to a gradual reduction in the habitat area suitable for *T. chinense*. The synergistic effects of climate change and land use change may limit the expansion of wild populations of *T. chinense*. In addition, the depletion of wild resources, coupled with excessive human mining, makes it difficult for the species to continue.

According to the above reasons, the wild resources of *T. chinense* have been unable to meet the drug demand, but due to its immature artificial breeding technology^20^, and China's national "Non-grain" policy prohibits the cultivation of cash crops on cultivated land. Therefore, the implementation of wild tend is the key to promote population recovery and sustainable utilization, which offer a viable solution to address the conflict between the scarcity of wild medicinal plant resources and the high market demand. Wild tending involves artificially or naturally increasing the population of targeted medicinal materials, resulting in significantly enhanced resource availability. It also helps maintain community balance after over-collection and ensures a continuous supply of high-quality medicinal materials^22^. The wild tending area has the characteristics of primitive environment, minimal human intervention, and distance from pollution sources. It is very important to predict the change range of the suitable habitat of *T. chinense* for formulating agricultural policies and planting planning^27^. Based on the current high suitable areas of *T. chinense* in China, combined with the future climate and land use change, this study planned the wild tending areas of *T. chinense* for hundred years. This approach effectively prevents significant economic losses caused by blind cultivation. Based on the results of the wild tending area analysis, we made the following recommendations: (1) In order to prevent the loss of germplasm resources caused by climate change and human activities, the collection and preservation of wild germplasm resources of *T. chinense* should be carried out in the climate transformation area. (2) In the region with stable climate, we should carry out the collection and evaluation of wild germplasm resources of *T. chinense*, comprehensively analyze the differences and correlations of its botanical traits, ecological traits, pharmacognostical traits and genetic traits, and build a comprehensive evaluation system of germplasm resources of *T. chinense*, so as to realize the effective protection and efficient utilization of resources (3) The precondition of artificial breeding and wild tending is a comprehensive understanding of the fundamental growth and development characteristics of TCM. Therefore, we should strengthen the research on population ecology and developmental biology, especially on the influence of different hosts on the composition and the mixed cultivation mode with other crops. This can not only maintain the stability of *T. chinense* population, but also improve the quality and yield of *T. chinense*. At the same time, the mixed cultivation mode can effectively make full use of land resources and make up for the shortage of grassland resources in some areas due to the dense population. (4) The local government should take more actions to protect the natural ecology, enhance the awareness of the protection and restoration of local natural land types, and avoid irreversible damage to them. (5) The planned wild tending area of *T. chinense* should be combined with local policies, especially the policies designated at the county level, integrate ecological, economic and social benefits, strengthen the establishment of wild tending medicinal material base, and effectively solve the contradiction between *T. chinense* resources and supply demand, ecological protection and biodiversity. At the same time, it is also necessary to strengthen the research on the base management mechanism to ensure the smooth operation of the base and achieve the purpose of wild tending.

## Conclusions

High-quality TCM should be planted in specific natural conditions. Therefore, predicting and evaluating the potential distribution areas for TCM species is crucial for the scientific planning of wild tending areas. The results show that although the stable area suitable for the growth of *T. chinense* is still large under climate change, the habitat area suitable for the growth of *T. chinense* is gradually shrinking, the environmental capacity is declining, and the population growth space is limited, which will seriously restrict the stability of its population distribution pattern and population number. This study has some implications for the study of global change, that is, when focusing on the changes in the distribution of species, we should not only pay attention to the changes in the upper climate, but also consider the impact of changes in land use patterns on biological populations in the under layer. This comprehensive consideration will more comprehensively reveal the response and adaptation strategies of ecosystems to global change. At present, our work is only carried out from a macro perspective. In the future work, we will continue to explore other factors that affect the change of the population structure of wild *T. chinense*, and look for the reasons for the change of the distribution pattern of wild population from the micro perspective. We should strengthen the research on the correlation between the content of effective components of *T. chinense* and environmental factors and hosts, and construct the spatial distribution layer of the content of effective components of *T. chinense*, so as to provide a more scientific and accurate theoretical basis for more reasonable planning of the wild tending area of *T. chinense*, which will provide more forward-looking and operable guidance for the protection and management of TCM, so as to ensure its sustainable utilization and promote the healthy development of the ecosystem.

### Supplementary Information

Supplementary Information 1.

Supplementary Information 2.

Supplementary Information 3.

## Data Availability

All data included in this study are available upon request by contact with the corresponding author.
