# Supplementary material for: Distribution and protection of Thesium chinense Turcz. under climate and land use change
Source: Sci Rep. 2024 Mar 18;14:6475. doi: 10.1038/s41598-024-57125-8 (PMC10948812; doi:10.1038/s41598-024-57125-8)
Supplement: Supplementary file 1 — Supplementary Information 1. [file 41598_2024_57125_MOESM1_ESM.docx]

# Biomod2 Run Code

## 1 Loading of relevant R packages

library(biomod2)

library(ggplot2)

library(gridExtra)

library(raster)

library(rasterVis)

## 2 Set work path

setwd("G:/Thesium_chinense/SDMs_modeling")

## 3 Reading species distribution data

Species_occ <- read.csv("G:/Thesium_chinense/occurence_data/spyhin_Thesium_chinense_thin1.csv")

head(Species_occ)

## 4 Read environment variables

current_envir <- raster::stack(

c(

bio08 = "G:/Thesium_chinense/SDMs_modeling/parameter_adjustment/tif/bio08.tif",

bio11 = "G:/Thesium_chinense/SDMs_modeling/parameter_adjustment/tif/bio11.tif",

bio15 = "G:/Thesium_chinense/SDMs_modeling/parameter_adjustment/tif/bio15.tif",

bio18 = "G:/Thesium_chinense/SDMs_modeling/parameter_adjustment/tif/bio18.tif",

bio19 = "G:/Thesium_chinense/SDMs_modeling/parameter_adjustment/tif/bio19.tif",

NDVI = "G:/Thesium_chinense/SDMs_modeling/parameter_adjustment/tif/ndvi.tif",

T_CLAY = "G:/Thesium_chinense/SDMs_modeling/parameter_adjustment/tif/t_clay.tif",

T_GRAVEL = "G:/Thesium_chinense/SDMs_modeling/parameter_adjustment/tif/t_gravel.tif",

T_SAND = "G:/Thesium_chinense/SDMs_modeling/parameter_adjustment/tif/t_sand.tif"

)

)

current_envir

## 5 Processing of species distribution data and generation of non distributed point data

Species_data <-

BIOMOD_FormatingData(

resp.var = Species_occ["SPEC"],

expl.var = current_envir,

resp.xy = Species_occ[,c("X","Y")],

resp.name ="Thesium_chinense",

PA.nb.rep = 3,

PA.nb.absences = 5000, #The number of non distributed points should be correspondingly reduced according to the scale, equal, 1000, 5000, 10000

PA.strategy = "random"

)

Species_data

plot(Species_data)

## 6 Setting of model parameters

Species_opt <-

BIOMOD_ModelingOptions(

GLM = NULL,

GBM = NULL,

GAM = NULL,

CTA = NULL,

ANN = NULL,

SRE = NULL,

FDA = NULL,

MARS = NULL,

RF = NULL,

MAXENT.Phillips = list(linear = TRUE,

quadratic = F,

product = TRUE,

threshold = TRUE,

hinge = F,

betamultiplier = 2,

maximumiterations = 5000)

)

## 7 Calculation and evaluation of single model

### 7.1 Operation of single model

Species_models <-

BIOMOD_Modeling(

data = Species_data,

models = c("GLM","GBM","GAM","CTA","ANN","SRE","FDA","MARS","RF","MAXENT.Phillips"),

models.options = Species_opt,

models.eval.meth = c('TSS', 'ROC'),

NbRunEval = 5, #Model repetitions

DataSplit = 70, #Training data percentage

VarImport = 3,#The number of permutations corresponding to the estimated importance of each variable

do.full.models = FALSE,

SaveObj = FALSE,

modeling.id = "Thesium_chinense"

)

Species_models

### 7.2 Evaluation score of single model

Species_models_scores <- get_evaluations(Species_models, as.data.frame = T)

write.csv(Species_models_scores, "./models_scores.csv")

## 8 Assessment of the Importance of Environmental Factors

### 8.1 Importance score of environmental factors

var_import <- get_variables_importance(Species_models, as.data.frame = TRUE)

varimp <- apply(var_import, c(1, 2), mean)

varimp

write.csv(var_import, "./Thesium_chinense_varimp_all.csv")

write.csv(varimp, "./Thesium_chinense_varimp_mean.csv")

## 9 Method and evaluation of ensemble model establishment

### 9.1 Establishment method

Species_ensemble_models <-

BIOMOD_EnsembleModeling(

modeling.output = Species_models,

chosen.models = "all",

em.by = "all",

eval.metric = c("TSS"),

eval.metric.quality.threshold = c(0.8),

models.eval.meth = c("TSS"),

prob.mean = T,

prob.cv = FALSE,

committee.averaging = F,

prob.mean.weight = FALSE,

VarImport = 0

)

### 9.2 Evaluation

Species_ensemble_models

Species_ensemble_models_scores <- get_evaluations(Species_ensemble_models)

Species_ensemble_models_scores

write.csv(Species_ensemble_models_scores, "./Thesium_chinense_ensemble_models_scores.csv")

## 10 Draw a distribution map of species under current climate conditions

### 10.1 Species distribution map of single model

Species_models_proj_current <-

BIOMOD_Projection(

modeling.output = Species_models,

new.env = current_envir,

proj.name = "current",

selected.models = 'all',

binary.meth = "TSS",

output.format = ".img",

do.stack = FALSE,

build.clamping.mask = FALSE

)

### 10.2 Operation of collective species distribution map

Species_ensemble_models_proj_current <-

BIOMOD_EnsembleForecasting(

EM.output = Species_ensemble_models,

projection.output = Species_models_proj_current,

binary.meth = "TSS",

output.format = ".img",

do.stack = FALSE

)

## 11 Draw a distribution map of species under future climate conditions

### 11.1 Average of three climate models

#### 11.1.2 2050s

##### 11.1.2.1 SSP126

future_venvir_2050s_SSP126 <-

stack(

c(

bio08 = "G:/Thesium_chinense/environment_data/Future/50126/bio08_50_126.tif",

bio11 = "G:/Thesium_chinense/environment_data/Future/50126/bio11_50_126.tif",

bio15 = "G:/Thesium_chinense/environment_data/Future/50126/bio15_50_126.tif",

bio18 = "G:/Thesium_chinense/environment_data/Future/50126/bio18_50_126.tif",

bio19 = "G:/Thesium_chinense/environment_data/Future/50126/bio19_50_126.tif",

NDVI = "G:/Thesium_chinense/SDMs_modeling/parameter_adjustment/tif/ndvi.tif",

T_CLAY = "G:/Thesium_chinense/SDMs_modeling/parameter_adjustment/tif/t_clay.tif",

T_GRAVEL = "G:/Thesium_chinense/SDMs_modeling/parameter_adjustment/tif/t_gravel.tif",

T_SAND = "G:/Thesium_chinense/SDMs_modeling/parameter_adjustment/tif/t_sand.tif"

))

future_venvir_2050s_SSP126

ProLau_models_proj_2050s_SSP126 <- BIOMOD_Projection(

modeling.output = Species_models,

proj.name = "2050s_SSP126",

new.env = future_venvir_2050s_SSP126,

metric.binary = "TSS",

do.stack = FALSE,

output.format = ".img")

ProLau_ensemble_models_proj_2050s_SSP126 <-

BIOMOD_EnsembleForecasting(

EM.output = Species_ensemble_models,

projection.output = ProLau_models_proj_2050s_SSP126,

metric.binary = "TSS",

do.stack = FALSE,

output.format = ".img")

##### 11.1.2.2 SSP245

future_venvir_2050s_SSP245 <- stack(

c(

bio08 = "G:/Thesium_chinense/environment_data/Future/50245/bio08_50_245.tif",

bio11 = "G:/Thesium_chinense/environment_data/Future/50245/bio11_50_245.tif",

bio15 = "G:/Thesium_chinense/environment_data/Future/50245/bio15_50_245.tif",

bio18 = "G:/Thesium_chinense/environment_data/Future/50245/bio18_50_245.tif",

bio19 = "G:/Thesium_chinense/environment_data/Future/50245/bio19_50_245.tif",

NDVI = "G:/Thesium_chinense/SDMs_modeling/parameter_adjustment/tif/ndvi.tif",

T_CLAY = "G:/Thesium_chinense/SDMs_modeling/parameter_adjustment/tif/t_clay.tif",

T_GRAVEL = "G:/Thesium_chinense/SDMs_modeling/parameter_adjustment/tif/t_gravel.tif",

T_SAND = "G:/Thesium_chinense/SDMs_modeling/parameter_adjustment/tif/t_sand.tif"

))

future_venvir_2050s_SSP245

ProLau_models_proj_2050s_SSP245 <- BIOMOD_Projection(

modeling.output = Species_models,

proj.name = "2050s_SSP245",

new.env = future_venvir_2050s_SSP245,

metric.binary = "TSS",

do.stack = FALSE,

output.format = ".img")

ProLau_ensemble_models_proj_2050s_SSP245 <-

BIOMOD_EnsembleForecasting(

EM.output = Species_ensemble_models,

projection.output = ProLau_models_proj_2050s_SSP245,

metric.binary = "TSS",

do.stack = FALSE,

output.format = ".img")

##### 11.1.2.3 SSP370

future_venvir_2050s_SSP370 <- stack(

c(

bio08 = "G:/Thesium_chinense/environment_data/Future/50370/bio08_50_370.tif",

bio11 = "G:/Thesium_chinense/environment_data/Future/50370/bio11_50_370.tif",

bio15 = "G:/Thesium_chinense/environment_data/Future/50370/bio15_50_370.tif",

bio18 = "G:/Thesium_chinense/environment_data/Future/50370/bio18_50_370.tif",

bio19 = "G:/Thesium_chinense/environment_data/Future/50370/bio19_50_370.tif",

NDVI = "G:/Thesium_chinense/SDMs_modeling/parameter_adjustment/tif/ndvi.tif",

T_CLAY = "G:/Thesium_chinense/SDMs_modeling/parameter_adjustment/tif/t_clay.tif",

T_GRAVEL = "G:/Thesium_chinense/SDMs_modeling/parameter_adjustment/tif/t_gravel.tif",

T_SAND = "G:/Thesium_chinense/SDMs_modeling/parameter_adjustment/tif/t_sand.tif"

)

)

future_venvir_2050s_SSP370

ProLau_models_proj_2050s_SSP370 <- BIOMOD_Projection(

modeling.output = Species_models,

proj.name = "2050s_SSP370",

new.env = future_venvir_2050s_SSP370,

metric.binary = "TSS",

do.stack = FALSE,

output.format = ".img")

ProLau_ensemble_models_proj_2050s_SSP370 <-

BIOMOD_EnsembleForecasting(

EM.output = Species_ensemble_models,

projection.output = ProLau_models_proj_2050s_SSP370,

metric.binary = "TSS",

do.stack = FALSE,

output.format = ".img")

##### 11.1.2.4 SSP585

future_venvir_2050s_SSP585 <- stack(

c(

bio08 = "G:/Thesium_chinense/environment_data/Future/50585/bio08_50_585.tif",

bio11 = "G:/Thesium_chinense/environment_data/Future/50585/bio11_50_585.tif",

bio15 = "G:/Thesium_chinense/environment_data/Future/50585/bio15_50_585.tif",

bio18 = "G:/Thesium_chinense/environment_data/Future/50585/bio18_50_585.tif",

bio19 = "G:/Thesium_chinense/environment_data/Future/50585/bio19_50_585.tif",

NDVI = "G:/Thesium_chinense/SDMs_modeling/parameter_adjustment/tif/ndvi.tif",

T_CLAY = "G:/Thesium_chinense/SDMs_modeling/parameter_adjustment/tif/t_clay.tif",

T_GRAVEL = "G:/Thesium_chinense/SDMs_modeling/parameter_adjustment/tif/t_gravel.tif",

T_SAND = "G:/Thesium_chinense/SDMs_modeling/parameter_adjustment/tif/t_sand.tif"

)

)

future_venvir_2050s_SSP585

ProLau_models_proj_2050s_SSP585 <- BIOMOD_Projection(

modeling.output = Species_models,

proj.name = "2050s_SSP585",

new.env = future_venvir_2050s_SSP585,

metric.binary = "TSS",

do.stack = FALSE,

output.format = ".img")

ProLau_ensemble_models_proj_2050s_SSP585 <-

BIOMOD_EnsembleForecasting(

EM.output = Species_ensemble_models,

projection.output = ProLau_models_proj_2050s_SSP585,

metric.binary = "TSS",

do.stack = FALSE,

output.format = ".img")

#### 11.1.3 2070s

##### 11.1.3.1 SSP126

future_venvir_2070s_SSP126 <-

stack(

c(

bio08 = "G:/Thesium_chinense/environment_data/Future/70126/bio08_70_126.tif",

bio11 = "G:/Thesium_chinense/environment_data/Future/70126/bio11_70_126.tif",

bio15 = "G:/Thesium_chinense/environment_data/Future/70126/bio15_70_126.tif",

bio18 = "G:/Thesium_chinense/environment_data/Future/70126/bio18_70_126.tif",

bio19 = "G:/Thesium_chinense/environment_data/Future/70126/bio19_70_126.tif",

NDVI = "G:/Thesium_chinense/SDMs_modeling/parameter_adjustment/tif/ndvi.tif",

T_CLAY = "G:/Thesium_chinense/SDMs_modeling/parameter_adjustment/tif/t_clay.tif",

T_GRAVEL = "G:/Thesium_chinense/SDMs_modeling/parameter_adjustment/tif/t_gravel.tif",

T_SAND = "G:/Thesium_chinense/SDMs_modeling/parameter_adjustment/tif/t_sand.tif"

))

future_venvir_2070s_SSP126

ProLau_models_proj_2070s_SSP126 <- BIOMOD_Projection(

modeling.output = Species_models,

proj.name = "2070s_SSP126",

new.env = future_venvir_2070s_SSP126,

metric.binary = "TSS",

do.stack = FALSE,

output.format = ".img")

ProLau_ensemble_models_proj_2070s_SSP126 <-

BIOMOD_EnsembleForecasting(

EM.output = Species_ensemble_models,

projection.output = ProLau_models_proj_2070s_SSP126,

metric.binary = "TSS",

do.stack = FALSE,

output.format = ".img")

##### 11.1.3.2 SSP245

future_venvir_2070s_SSP245 <- stack(

c(

bio08 = "G:/Thesium_chinense/environment_data/Future/70245/bio08_70_245.tif",

bio11 = "G:/Thesium_chinense/environment_data/Future/70245/bio11_70_245.tif",

bio15 = "G:/Thesium_chinense/environment_data/Future/70245/bio15_70_245.tif",

bio18 = "G:/Thesium_chinense/environment_data/Future/70245/bio18_70_245.tif",

bio19 = "G:/Thesium_chinense/environment_data/Future/70245/bio19_70_245.tif",

NDVI = "G:/Thesium_chinense/SDMs_modeling/parameter_adjustment/tif/ndvi.tif",

T_CLAY = "G:/Thesium_chinense/SDMs_modeling/parameter_adjustment/tif/t_clay.tif",

T_GRAVEL = "G:/Thesium_chinense/SDMs_modeling/parameter_adjustment/tif/t_gravel.tif",

T_SAND = "G:/Thesium_chinense/SDMs_modeling/parameter_adjustment/tif/t_sand.tif"

))

future_venvir_2070s_SSP245

ProLau_models_proj_2070s_SSP245 <- BIOMOD_Projection(

modeling.output = Species_models,

proj.name = "2070s_SSP245",

new.env = future_venvir_2070s_SSP245,

metric.binary = "TSS",

do.stack = FALSE,

output.format = ".img")

ProLau_ensemble_models_proj_2070s_SSP245 <-

BIOMOD_EnsembleForecasting(

EM.output = Species_ensemble_models,

projection.output = ProLau_models_proj_2070s_SSP245,

metric.binary = "TSS",

do.stack = FALSE,

output.format = ".img")

##### 11.1.3.3 SSP370

future_venvir_2070s_SSP370 <- stack(

c(

bio08 = "G:/Thesium_chinense/environment_data/Future/70370/bio08_70_370.tif",

bio11 = "G:/Thesium_chinense/environment_data/Future/70370/bio11_70_370.tif",

bio15 = "G:/Thesium_chinense/environment_data/Future/70370/bio15_70_370.tif",

bio18 = "G:/Thesium_chinense/environment_data/Future/70370/bio18_70_370.tif",

bio19 = "G:/Thesium_chinense/environment_data/Future/70370/bio19_70_370.tif",

NDVI = "G:/Thesium_chinense/SDMs_modeling/parameter_adjustment/tif/ndvi.tif",

T_CLAY = "G:/Thesium_chinense/SDMs_modeling/parameter_adjustment/tif/t_clay.tif",

T_GRAVEL = "G:/Thesium_chinense/SDMs_modeling/parameter_adjustment/tif/t_gravel.tif",

T_SAND = "G:/Thesium_chinense/SDMs_modeling/parameter_adjustment/tif/t_sand.tif"

))

future_venvir_2070s_SSP370

ProLau_models_proj_2070s_SSP370 <- BIOMOD_Projection(

modeling.output = Species_models,

proj.name = "2070s_SSP370",

new.env = future_venvir_2070s_SSP370,

metric.binary = "TSS",

do.stack = FALSE,

output.format = ".img")

ProLau_ensemble_models_proj_2070s_SSP370 <-

BIOMOD_EnsembleForecasting(

EM.output = Species_ensemble_models,

projection.output = ProLau_models_proj_2070s_SSP370,

metric.binary = "TSS",

do.stack = FALSE,

output.format = ".img")

##### 11.1.3.4 SSP585

future_venvir_2070s_SSP585 <- stack(

c(

bio08 = "G:/Thesium_chinense/environment_data/Future/70585/bio08_70_585.tif",

bio11 = "G:/Thesium_chinense/environment_data/Future/70585/bio11_70_585.tif",

bio15 = "G:/Thesium_chinense/environment_data/Future/70585/bio15_70_585.tif",

bio18 = "G:/Thesium_chinense/environment_data/Future/70585/bio18_70_585.tif",

bio19 = "G:/Thesium_chinense/environment_data/Future/70585/bio19_70_585.tif",

NDVI = "G:/Thesium_chinense/SDMs_modeling/parameter_adjustment/tif/ndvi.tif",

T_CLAY = "G:/Thesium_chinense/SDMs_modeling/parameter_adjustment/tif/t_clay.tif",

T_GRAVEL = "G:/Thesium_chinense/SDMs_modeling/parameter_adjustment/tif/t_gravel.tif",

T_SAND = "G:/Thesium_chinense/SDMs_modeling/parameter_adjustment/tif/t_sand.tif"

))

future_venvir_2070s_SSP585

ProLau_models_proj_2070s_SSP585 <- BIOMOD_Projection(

modeling.output = Species_models,

proj.name = "2070s_SSP585",

new.env = future_venvir_2070s_SSP585,

metric.binary = "TSS",

do.stack = FALSE,

output.format = ".img")

ProLau_ensemble_models_proj_2070s_SSP585 <-

BIOMOD_EnsembleForecasting(

EM.output = Species_ensemble_models,

projection.output = ProLau_models_proj_2070s_SSP585,

metric.binary = "TSS",

do.stack = FALSE,

output.format = ".img")

#### 11.1.4 2090s

##### 11.1.4.1 SSP126

future_venvir_2090s_SSP126 <-

stack(

c(

bio08 = "G:/Thesium_chinense/environment_data/Future/90126/bio08_90_126.tif",

bio11 = "G:/Thesium_chinense/environment_data/Future/90126/bio11_90_126.tif",

bio15 = "G:/Thesium_chinense/environment_data/Future/90126/bio15_90_126.tif",

bio18 = "G:/Thesium_chinense/environment_data/Future/90126/bio18_90_126.tif",

bio19 = "G:/Thesium_chinense/environment_data/Future/90126/bio19_90_126.tif",

NDVI = "G:/Thesium_chinense/SDMs_modeling/parameter_adjustment/tif/ndvi.tif",

T_CLAY = "G:/Thesium_chinense/SDMs_modeling/parameter_adjustment/tif/t_clay.tif",

T_GRAVEL = "G:/Thesium_chinense/SDMs_modeling/parameter_adjustment/tif/t_gravel.tif",

T_SAND = "G:/Thesium_chinense/SDMs_modeling/parameter_adjustment/tif/t_sand.tif"

))

future_venvir_2090s_SSP126

ProLau_models_proj_2090s_SSP126 <- BIOMOD_Projection(

modeling.output = Species_models,

proj.name = "2090s_SSP126",

new.env = future_venvir_2090s_SSP126,

metric.binary = "TSS",

do.stack = FALSE,

output.format = ".img")

ProLau_ensemble_models_proj_2090s_SSP126 <-

BIOMOD_EnsembleForecasting(

EM.output = Species_ensemble_models,

projection.output = ProLau_models_proj_2090s_SSP126,

metric.binary = "TSS",

do.stack = FALSE,

output.format = ".img")

##### 11.1.4.2 SSP245

future_venvir_2090s_SSP245 <- stack(

c(

bio08 = "G:/Thesium_chinense/environment_data/Future/90245/bio08_90_245.tif",

bio11 = "G:/Thesium_chinense/environment_data/Future/90245/bio11_90_245.tif",

bio15 = "G:/Thesium_chinense/environment_data/Future/90245/bio15_90_245.tif",

bio18 = "G:/Thesium_chinense/environment_data/Future/90245/bio18_90_245.tif",

bio19 = "G:/Thesium_chinense/environment_data/Future/90245/bio19_90_245.tif",

NDVI = "G:/Thesium_chinense/SDMs_modeling/parameter_adjustment/tif/ndvi.tif",

T_CLAY = "G:/Thesium_chinense/SDMs_modeling/parameter_adjustment/tif/t_clay.tif",

T_GRAVEL = "G:/Thesium_chinense/SDMs_modeling/parameter_adjustment/tif/t_gravel.tif",

T_SAND = "G:/Thesium_chinense/SDMs_modeling/parameter_adjustment/tif/t_sand.tif"

))

future_venvir_2090s_SSP245

ProLau_models_proj_2090s_SSP245 <- BIOMOD_Projection(

modeling.output = Species_models,

proj.name = "2090s_SSP245",

new.env = future_venvir_2090s_SSP245,

metric.binary = "TSS",

do.stack = FALSE,

output.format = ".img")

ProLau_ensemble_models_proj_2090s_SSP245 <-

BIOMOD_EnsembleForecasting(

EM.output = Species_ensemble_models,

projection.output = ProLau_models_proj_2090s_SSP245,

metric.binary = "TSS",

do.stack = FALSE,

output.format = ".img")

##### 11.1.4.3 SSP370

future_venvir_2090s_SSP370 <- stack(

c(

bio08 = "G:/Thesium_chinense/environment_data/Future/90370/bio08_90_370.tif",

bio11 = "G:/Thesium_chinense/environment_data/Future/90370/bio11_90_370.tif",

bio15 = "G:/Thesium_chinense/environment_data/Future/90370/bio15_90_370.tif",

bio18 = "G:/Thesium_chinense/environment_data/Future/90370/bio18_90_370.tif",

bio19 = "G:/Thesium_chinense/environment_data/Future/90370/bio19_90_370.tif",

NDVI = "G:/Thesium_chinense/SDMs_modeling/parameter_adjustment/tif/ndvi.tif",

T_CLAY = "G:/Thesium_chinense/SDMs_modeling/parameter_adjustment/tif/t_clay.tif",

T_GRAVEL = "G:/Thesium_chinense/SDMs_modeling/parameter_adjustment/tif/t_gravel.tif",

T_SAND = "G:/Thesium_chinense/SDMs_modeling/parameter_adjustment/tif/t_sand.tif"

))

future_venvir_2090s_SSP370

ProLau_models_proj_2090s_SSP370 <- BIOMOD_Projection(

modeling.output = Species_models,

proj.name = "2090s_SSP370",

new.env = future_venvir_2090s_SSP370,

metric.binary = "TSS",

do.stack = FALSE,

output.format = ".img")

ProLau_ensemble_models_proj_2090s_SSP370 <-

BIOMOD_EnsembleForecasting(

EM.output = Species_ensemble_models,

projection.output = ProLau_models_proj_2090s_SSP370,

metric.binary = "TSS",

do.stack = FALSE,

output.format = ".img")

##### 11.1.4.4 SSP585

future_venvir_2090s_SSP585 <- stack(

c(

bio08 = "G:/Thesium_chinense/environment_data/Future/90585/bio08_90_585.tif",

bio11 = "G:/Thesium_chinense/environment_data/Future/90585/bio11_90_585.tif",

bio15 = "G:/Thesium_chinense/environment_data/Future/90585/bio15_90_585.tif",

bio18 = "G:/Thesium_chinense/environment_data/Future/90585/bio18_90_585.tif",

bio19 = "G:/Thesium_chinense/environment_data/Future/90585/bio19_90_585.tif",

NDVI = "G:/Thesium_chinense/SDMs_modeling/parameter_adjustment/tif/ndvi.tif",

T_CLAY = "G:/Thesium_chinense/SDMs_modeling/parameter_adjustment/tif/t_clay.tif",

T_GRAVEL = "G:/Thesium_chinense/SDMs_modeling/parameter_adjustment/tif/t_gravel.tif",

T_SAND = "G:/Thesium_chinense/SDMs_modeling/parameter_adjustment/tif/t_sand.tif"

))

future_venvir_2090s_SSP585

ProLau_models_proj_2090s_SSP585 <- BIOMOD_Projection(

modeling.output = Species_models,

proj.name = "2090s_SSP585",

new.env = future_venvir_2090s_SSP585,

metric.binary = "TSS",

do.stack = FALSE,

output.format = ".img")

ProLau_ensemble_models_proj_2090s_SSP585 <-

BIOMOD_EnsembleForecasting(

EM.output = Species_ensemble_models,

projection.output = ProLau_models_proj_2090s_SSP585,

metric.binary = "TSS",

do.stack = FALSE,

output.format = ".img")
