# Supplementary material for: Distribution and protection of Thesium chinense Turcz. under climate and land use change
Source: Sci Rep. 2024 Mar 18;14:6475. doi: 10.1038/s41598-024-57125-8 (PMC10948812; doi:10.1038/s41598-024-57125-8)
Supplement: Supplementary file 2 — Supplementary Information 2. [file 41598_2024_57125_MOESM2_ESM.docx]

install.packages("devtools")

devtools::install_github("marlonecobos/kuenm")

library(kuenm)

library(raster)

library(rgeos)

setwd("G:/Thesium_chinense/SDMs_modeling/maxent_parameter")

poly <- readWKT("POLYGON((

-180.0 81.29,

180.0 81.29,

180.0 -10.93,

-180.0 -10.93,

-180.0 81.29

))", p4s = CRS("+proj=longlat +datum=WGS84"))

inputDir <- "./tif"

outputDir <- "./asc_R2"

dir.create(outputDir)

files <- list.files(inputDir, pattern = '.tif$', full.names = TRUE)

for (i in 1:length(files)) {

cat(i, ' ')

r <- raster(files[i])

r <- crop(r, poly)

outfile <- paste0(outputDir, '/', names(r), '.asc')

writeRaster(r, filename = outfile, format = 'ascii', overwrite = TRUE)}

occs <- read.csv("spyhin_Thesium_chinense_thin1.csv")

set.seed(1)

split <- kuenm_occsplit(

occ = occs,

train.proportion = 0.75,

method = "random",

save = TRUE,

name = "T.chinense")

occ_joint <- "T.chinense_joint.csv"

occ_tra <- "T.chinense_train.csv"

M_var_dir <- "Environ_variables"

batch_cal <- "Candidate_models"

out_dir <- "Candidate_models"

reg_mult <- c(seq(0.1, 1, 0.1),seq(2,6,1),8,10)

f_clas <- "all"

args <- NULL

maxent_path <- "G:/Thesium_chinense/SDMs_modeling/maxent_parameter"

wait <- FALSE

run <- TRUE

kuenm_cal(

occ.joint = occ_joint,

occ.tra = occ_tra,

M.var.dir = M_var_dir,

batch = batch_cal,

out.dir = out_dir,

reg.mult = reg_mult,

f.clas = f_clas,

args = args,

maxent.path = maxent_path,

wait = wait,

run = run)

test <- "D.daT.chinense_test.csvt_ev"

oual <- "Calibration_Results"

threshold <- 5

rand_percent <- 50

iterations <- 100

kept <- TRUE

selection <- "OR_AICc"

parallel_proc <- FALSE

cal_eval <- kuenm_ceval(

path = out_dir,

occ.joint = occ_joint,

occ.tra = occ_tra,

occ.test = occ_test,

batch = batch_cal,

out.eval = out_eval,

threshold = threshold,

rand.percent = rand_percent,

iterations = iterations,

kept = kept,

selection = selection,

parallel.proc = parallel_proc)
