# Supplementary material for: Distribution and protection of Thesium chinense Turcz. under climate and land use change
Source: Sci Rep. 2024 Mar 18;14:6475. doi: 10.1038/s41598-024-57125-8 (PMC10948812; doi:10.1038/s41598-024-57125-8)
Supplement: Supplementary file 3 — Supplementary Information 3. [file 41598_2024_57125_MOESM3_ESM.docx]

**Table. S1** Environmental factors used in species distribution modeling.

| **Classification** | **Variable abbreviation** | **Name** | **Unit** |
| --- | --- | --- | --- |
| Climate | Bio01 | annual mean temperature | ℃ |
| Climate | Bio02 | mean diurnal range | ℃ |
| Climate | Bio03 | isothermally | - |
| Climate | Bio04 | temperature seasonality |  |
| Climate | Bio05 | maximum temperature of warmest month | ℃ |
| Climate | Bio06 | minimum temperature of coldest month | ℃ |
| Climate | Bio07 | temperature annual range | ℃ |
| Climate | Bio08 | mean temperature of wettest quarter | ℃ |
| Climate | Bio09 | mean temperature of driest quarter | ℃ |
| Climate | Bio10 | mean temperature of warmest quarter | ℃ |
| Climate | Bio11 | mean temperature of coldest quarter | ℃ |
| Climate | Bio12 | annual precipitation | mm |
| Climate | Bio13 | precipitation of wettest period | mm |
| Climate | Bio14 | precipitation of driest period | mm |
| Climate | Bio15 | precipitation seasonality CV | - |
| Climate | Bio16 | precipitation of wettest quarter | mm |
| Climate | Bio17 | precipitation of driest quarter | mm |
| Climate | Bio18 | precipitation of warmest quarter | mm |
| Climate | Bio19 | precipitation of coldest quarter | mm |
| Biology | NDVI | normalized difference vegetation index | - |
| Soil | T_OC | Topsoil organic carbon | % |
| Soil | T_pH_H_2_O | Topsoil pH(H_2_O) | -log(H^+^) |
| Soil | T_Clay | Topsoil clay content | % |
| Soil | T_Gravel | Topsoil gravel content | % |
| Soil | T_Sand | Topsoil sand content | % |

**Table. S2** Classification system table of China's National Land Use and Cover Change.

| **First level type** | | **Secondary type** | |
| --- | --- | --- | --- |
| **number** | **name** | **number** | **name** |
| 1 | Cultivated land |  |  |
|  |  | 11 | Paddy field |
|  |  | 12 | Dry land |
| 2 | Forest |  |  |
|  |  | 21 | Forestland |
|  |  | 22 | Shrub |
|  |  | 23 | Sparse woods |
|  |  | 24 | Other woodland |
| 3 | Grassland |  |  |
|  |  | 31 | High coverage grassland |
|  |  | 32 | Moderate coverage grassland |
|  |  | 33 | Low coverage grassland |
| 4 | Waters |  |  |
|  |  | 41 | River and canal |
|  |  | 42 | Lake |
|  |  | 43 | Reservoir and pond |
|  |  | 44 | Permanent glacier snow land |
|  |  | 45 | Mudflat |
|  |  | 46 | Shoal |
| 5 | Construction land |  |  |
|  |  | 51 | Urban land |
|  |  | 52 | Rural settlement |
|  |  | 53 | Other construction land |
| 6 | Unused land |  |  |
|  |  | 61 | Sand |
|  |  | 62 | Gobi |
|  |  | 63 | Saline-alkali soil |
|  |  | 64 | Swampy land |
|  |  | 65 | Bare land |
|  |  | 66 | Bare rocky ground |
|  |  | 67 | other |


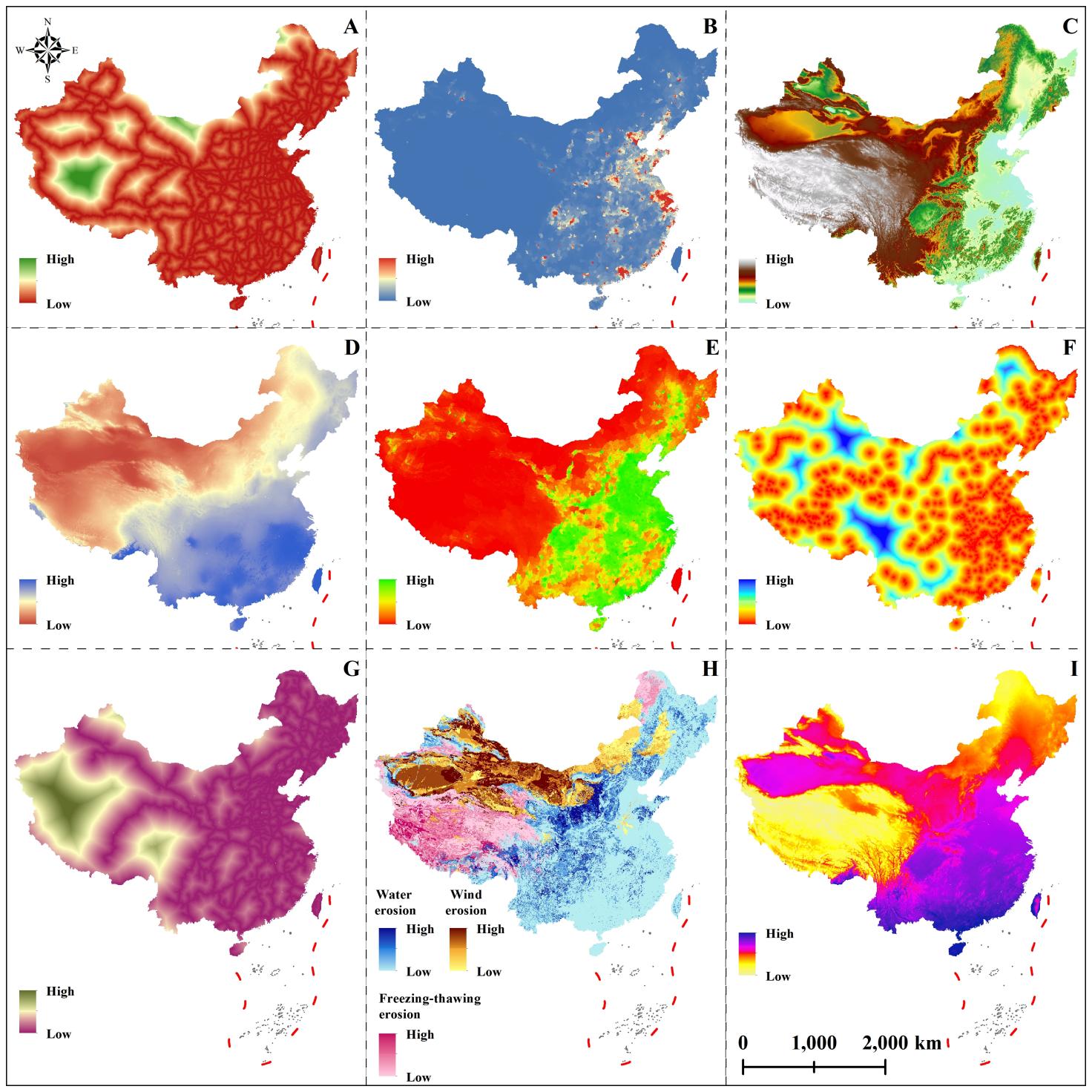


**Figure. S1** Drivers factors of land use. (A): Distance from road; (B): GDP; (C): Altitude; (D): Annual precipitation; (E): Population density; (F): Distance from waters; (G): Distance from railway; (H): Soil erosion degree; (I): Annual mean temperature.

**Table. S3** Neighborhood weight of various land use types.

| **Land use type** | **Cultivated land** | **Forest** | **Grassland** | **Waters** | **Construction land** | **Unused land** |
| --- | --- | --- | --- | --- | --- | --- |
| **Neighborhood weight** | 0.034 | 0.025 | 0.029 | 0.138 | 1 | 0.01 |

**Table. S4** Land use transfer matrix.

|  | | **Land use in 2015** | | | | | |
| --- | --- | --- | --- | --- | --- | --- | --- |
|  |  | **Cultivated land** | **Forest** | **Grassland** | **Waters** | **Construction land** | **Unused land** |
| **Land use in 2010** | **Cultivated land** | 1 | 1 | 1 | 1 | 1 | 0 |
|  | **Forest** | 1 | 1 | 0 | 0 | 0 | 0 |
|  | **Grassland** | 1 | 1 | 1 | 1 | 1 | 0 |
|  | **Waters** | 1 | 0 | 0 | 1 | 1 | 0 |
|  | **Construction land** | 1 | 0 | 0 | 0 | 1 | 0 |
|  | **Unused land** | 1 | 1 | 1 | 1 | 1 | 1 |

**Table. S5** All environmental factors used to build the model and their importance.

| **Environment variable** | **Model** | | | | | | | | | | |
| --- | --- | --- | --- | --- | --- | --- | --- | --- | --- | --- | --- |
|  | **ANN** | **CTA** | **FDA** | **GAM** | **GBM** | **GLM** | **MARS** | **MAXENT** | **RF** | **SRE** | **Average** |
| Bio18 | 0.82 | 0.71 | 0.23 | 0.37 | 0.56 | 0.44 | 0.31 | 0.33 | 0.35 | 0.55 | **0.47** |
| Bio11 | 0.36 | 0.43 | 0.34 | 0.37 | 0.27 | 0.25 | 0.24 | 0.41 | 0.24 | 0.37 | **0.33** |
| Bio08 | 0.23 | 0.28 | 0.22 | 0.20 | 0.14 | 0.18 | 0.16 | 0.13 | 0.19 | 0.35 | **0.21** |
| Bio15 | 0.20 | 0.00 | 0.03 | 0.15 | 0.03 | 0.20 | 0.00 | 0.16 | 0.06 | 0.30 | 0.11 |
| Bio19 | 0.33 | 0.04 | 0.02 | 0.22 | 0.03 | 0.03 | 0.20 | 0.03 | 0.12 | 0.10 | 0.11 |
| NDVI | 0.02 | 0.01 | 0.01 | 0.21 | 0.03 | 0.07 | 0.12 | 0.11 | 0.09 | 0.29 | 0.09 |
| T_Sand | 0.14 | 0.00 | 0.00 | 0.10 | 0.00 | 0.00 | 0.01 | 0.04 | 0.04 | 0.10 | 0.04 |
| T_Clay | 0.08 | 0.01 | 0.00 | 0.20 | 0.01 | 0.00 | 0.00 | 0.01 | 0.02 | 0.06 | 0.04 |
| T_Gravel | 0.06 | 0.00 | 0.00 | 0.07 | 0.00 | 0.01 | 0.02 | 0.01 | 0.01 | 0.11 | 0.03 |

**Table. S6** Numerical changes of main environmental factors in the current distribution area of *T. chinense* under the combination of future climate scenarios.

| **Environment variable** | **Climatic scenario** | | **95% quantiles** | | **Average** |
| --- | --- | --- | --- | --- | --- |
|  |  |  | **2.5%** | **97.5%** |  |
| Bio18/mm | Current | - | 216.27 | 741.47 | 415.60 |
|  | 2050s | SSP1-2.6 | 250.96 | 801.14 | 451.72 |
|  |  | SSP2-4.5 | 247.88 | 775.54 | 443.09 |
|  |  | SSP3-7.0 | 244.53 | 761.15 | 435.94 |
|  |  | SSP5-8.5 | 252.48 | 786.92 | 444.73 |
|  | 2070s | SSP1-2.6 | 252.70 | 806.21 | 453.98 |
|  |  | SSP2-4.5 | 253.58 | 780.52 | 448.09 |
|  |  | SSP3-7.0 | 248.82 | 770.24 | 439.85 |
|  |  | SSP5-8.5 | 253.92 | 789.84 | 447.93 |
|  | 2090s | SSP1-2.6 | 254.74 | 807.17 | 454.86 |
|  |  | SSP2-4.5 | 251.63 | 796.04 | 452.00 |
|  |  | SSP3-7.0 | 252.05 | 772.32 | 437.68 |
|  |  | SSP5-8.5 | 261.25 | 806.45 | 450.57 |
| Bio11/℃ | Current | - | -25.03 | 11.62 | -4.44 |
|  | 2050s | SSP1-2.6 | -22.14 | 13.18 | -2.24 |
|  |  | SSP2-4.5 | -21.65 | 13.13 | -2.12 |
|  |  | SSP3-7.0 | -21.56 | 12.98 | -2.19 |
|  |  | SSP5-8.5 | -20.96 | 13.65 | -1.52 |
|  | 2070s | SSP1-2.6 | -22.24 | 13.31 | -2.23 |
|  |  | SSP2-4.5 | -21.06 | 13.56 | -1.61 |
|  |  | SSP3-7.0 | -20.27 | 13.92 | -1.08 |
|  |  | SSP5-8.5 | -19.23 | 14.88 | -0.11 |
|  | 2090s | SSP1-2.6 | -22.15 | 13.17 | -2.30 |
|  |  | SSP2-4.5 | -20.67 | 14.09 | -1.15 |
|  |  | SSP3-7.0 | -18.77 | 14.79 | 0.04 |
|  |  | SSP5-8.5 | -16.95 | 16.41 | 1.77 |
| Bio08/℃ | Current | - | 9.80 | 26.33 | 20.33 |
|  | 2050s | SSP1-2.6 | 12.02 | 29.38 | 23.11 |
|  |  | SSP2-4.5 | 12.20 | 29.51 | 23.21 |
|  |  | SSP3-7.0 | 12.22 | 29.10 | 23.10 |
|  |  | SSP5-8.5 | 12.66 | 29.78 | 23.72 |
|  | 2070s | SSP1-2.6 | 12.09 | 29.46 | 23.19 |
|  |  | SSP2-4.5 | 12.69 | 29.98 | 23.72 |
|  |  | SSP3-7.0 | 12.99 | 30.04 | 23.96 |
|  |  | SSP5-8.5 | 13.75 | 31.01 | 24.95 |
|  | 2090s | SSP1-2.6 | 12.02 | 29.40 | 23.10 |
|  |  | SSP2-4.5 | 13.01 | 30.13 | 24.08 |
|  |  | SSP3-7.0 | 13.99 | 30.73 | 24.95 |
|  |  | SSP5-8.5 | 15.16 | 32.19 | 26.28 |

**
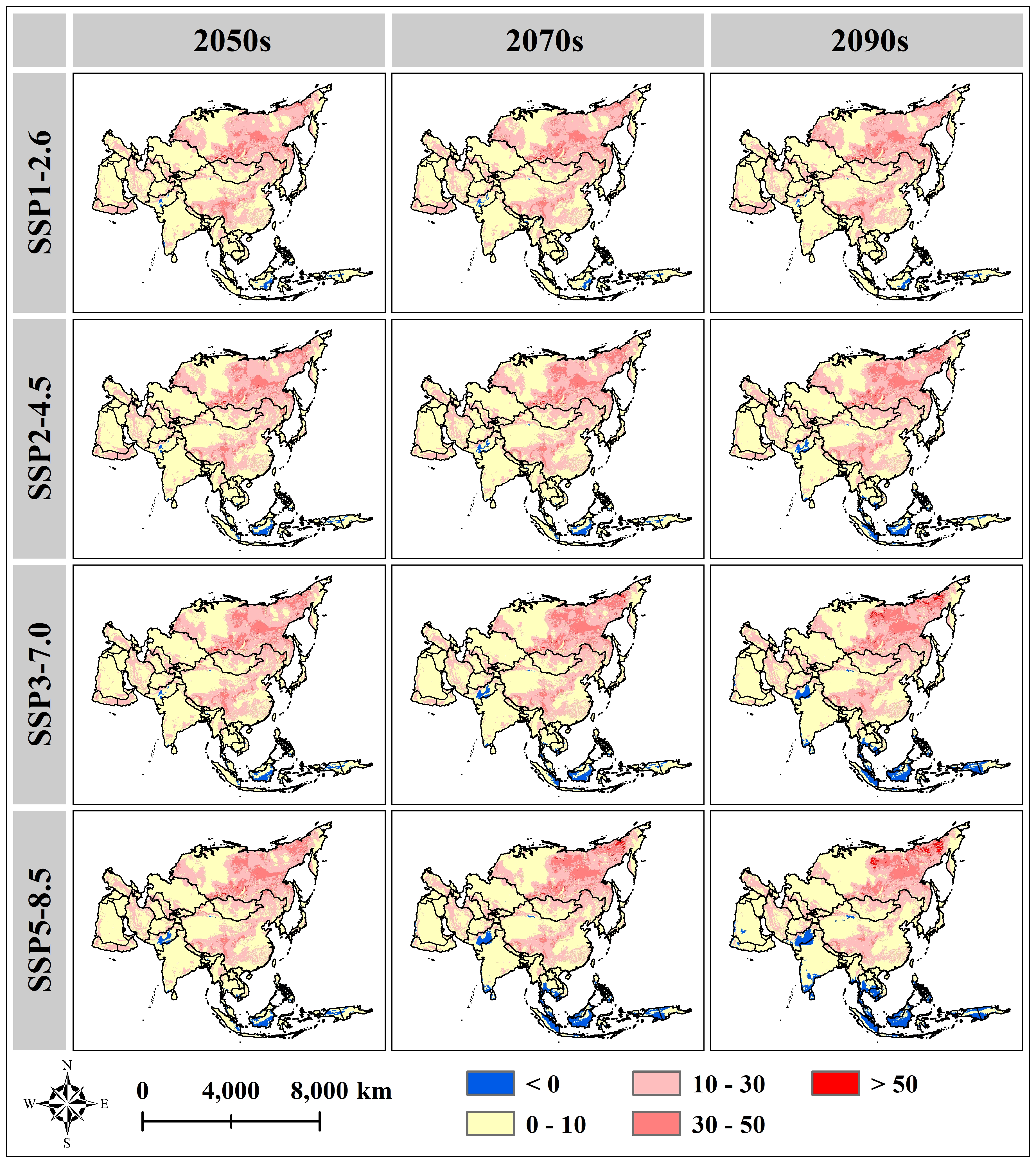
**

**Figure. S2** MESS analysis of the study area in the future.


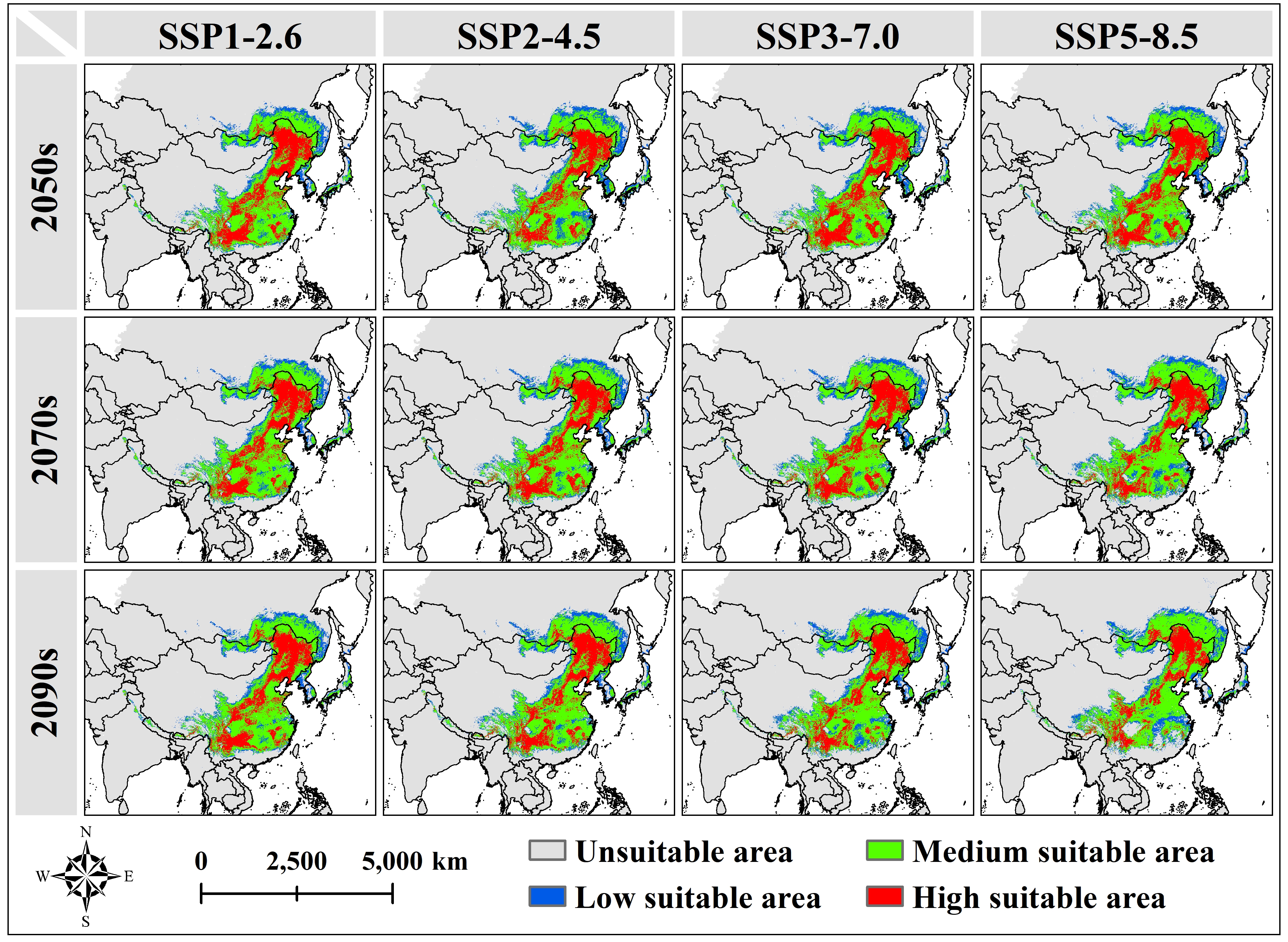


**Figure. S3** Spatial distribution map of the complete niche of *T. chinense*.





**Figure. S4** Area change map of the complete niche of *T. chinense*.


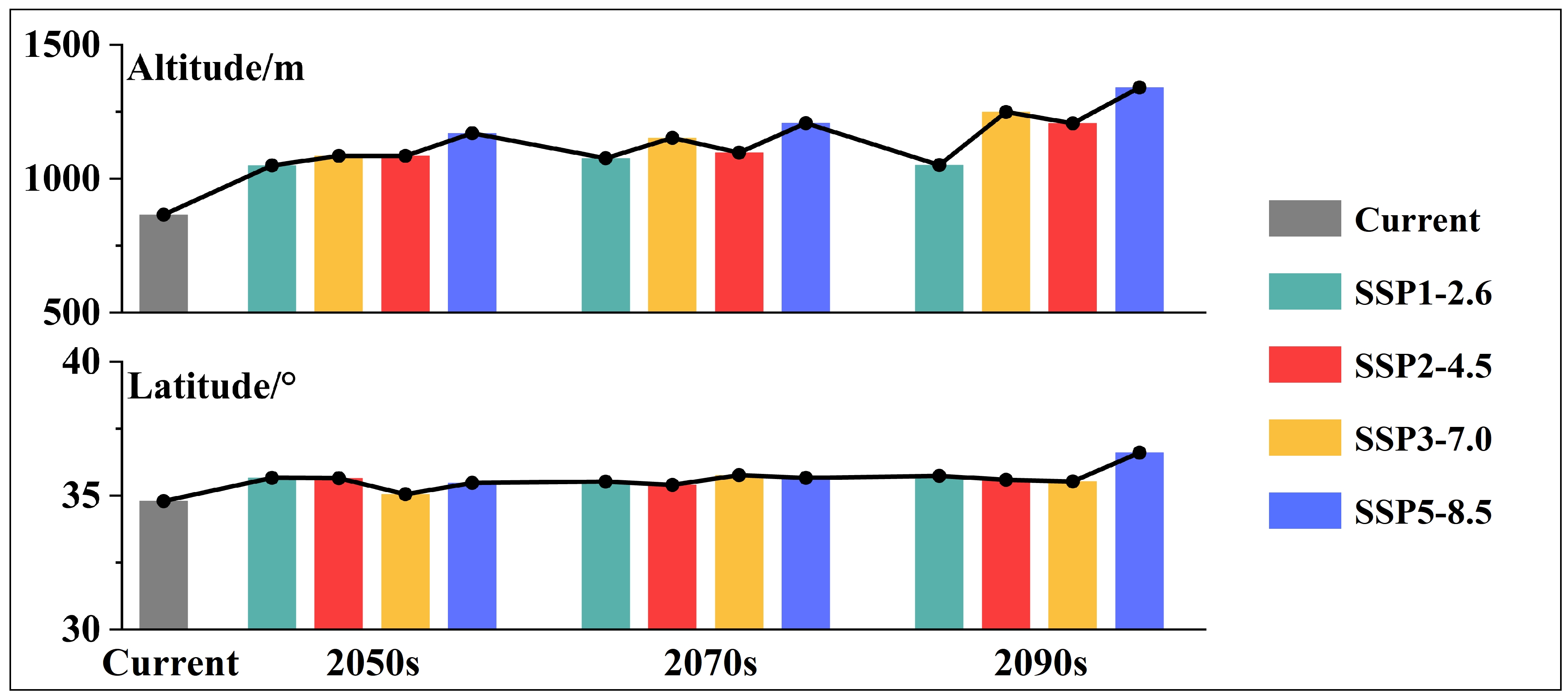


**Figure. S5** Changes in altitude and latitude of suitable areas of *T. chinense* in China.

**Table. S7** Contribution of driving factors affecting land use type change.

| **Contribution** | **Land use** | | | | | |
| --- | --- | --- | --- | --- | --- | --- |
|  | **Cultivated land** | **Forests** | **Grassland** | **Waters** | **Construction land** | **Unused land** |
| **Distance from road** | 0.00 | 0.03 | 0.17 | 0.01 | 0.13 | 0.04 |
| **GDP** | 0.17 | 0.09 | 0.05 | 0.05 | 0.15 | 0.04 |
| **Altitude** | 0.17 | -0.01 | 0.09 | 0.10 | 0.10 | **0.65** |
| **Precipitation** | **0.38** | 0.05 | 0.17 | 0.00 | 0.03 | 0.14 |
| **Population density** | 0.04 | 0.13 | 0.03 | 0.15 | **0.36** | 0.10 |
| **Distance from waters** | 0.08 | 0.08 | **0.33** | **0.50** | 0.12 | 0.00 |
| **Distance from railway** | 0.02 | 0.19 | 0.04 | 0.00 | 0.03 | 0.00 |
| **Soil erosion degree** | 0.00 | 0.00 | 0.00 | 0.00 | 0.00 | 0.01 |
| **temperature** | 0.13 | **0.42** | 0.12 | 0.21 | 0.07 | 0.01 |

**
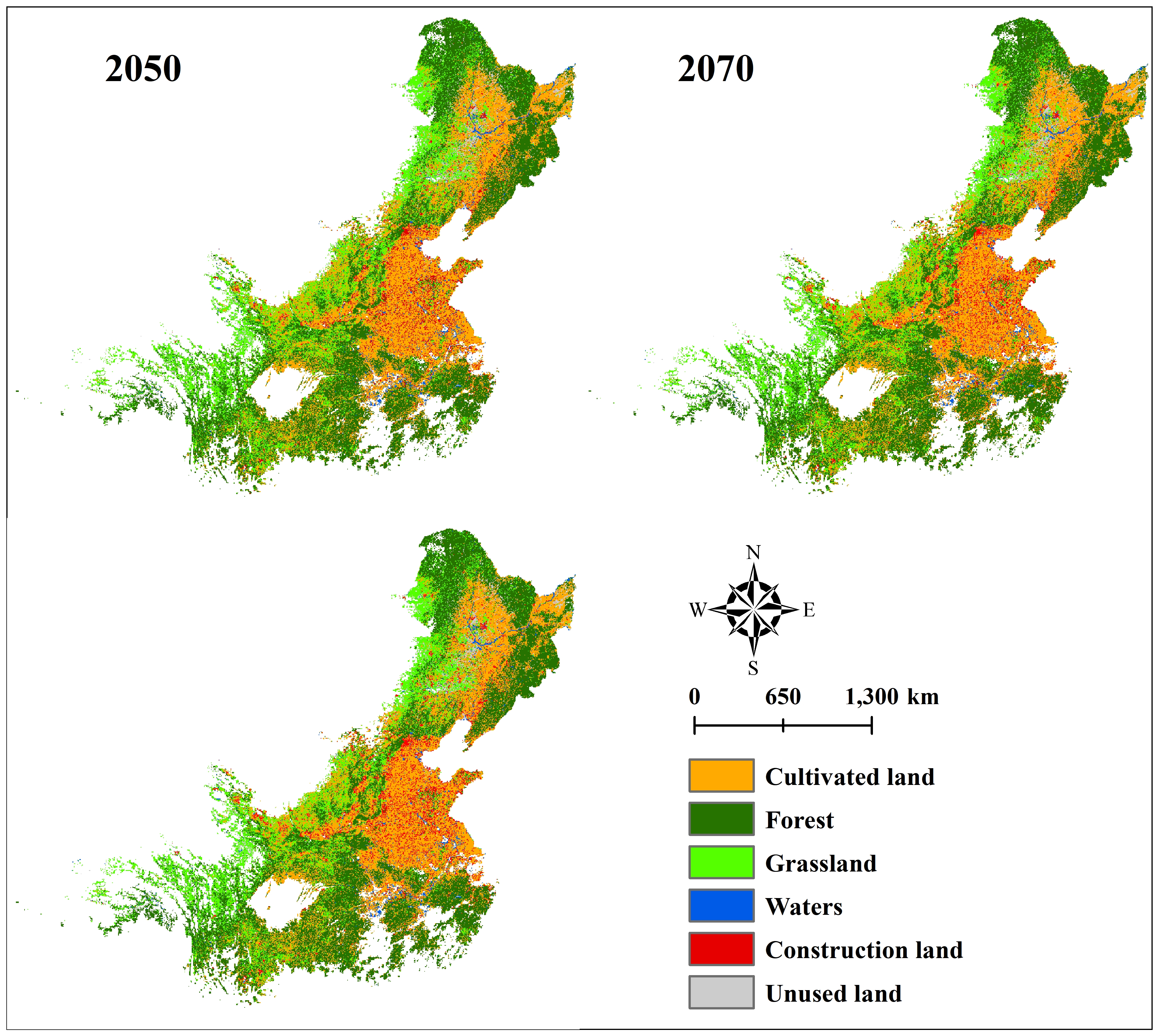
**

**Figure. S6** Land use distribution pattern in climate stable area of *T. chinense* in China in 2050, 2070, and 2090.

**Table. S8** Transfer areas of *T. chinense* among land use types in climate stable areas of China from 2015 to 2050, 2050 to 2070, and 2070 to 2090.

| **Years** |  | **Change to 2050** | | | | | |
| --- | --- | --- | --- | --- | --- | --- | --- |
|  | **Area/(km^2^)** | **Cultivated land** | **Forests** | **Grassland** | **Waters** | **Construction land** | **Unused land** |
| **2015** | **Cultivated land** | 1247050 | 1800 | 1750 | 400 | 19100 | 925 |
|  | **Forests** | 750 | 1439425 | 425 | 50 | 225 | 0 |
|  | **Grassland** | 525 | 22475 | 732875 | 550 | 30700 | 375 |
|  | **Waters** | 825 | 275 | 225 | 78525 | 100 | 200 |
|  | **Construction land** | 8900 | 1775 | 975 | 300 | 132275 | 275 |
|  | **Unused land** | 200 | 150 | 4475 | 75 | 1100 | 94475 |
|  |  | **Change to 2070** | | | | | |
|  | **Area/(km^2^)** | **Cultivated land** | **Forests** | **Grassland** | **Waters** | **Construction land** | **Unused land** |
| **2050** | **Cultivated land** | 1234425 | 0 | 0 | 0 | 23825 | 0 |
|  | **Forests** | 0 | 1453275 | 12475 | 0 | 100 | 50 |
|  | **Grassland** | 0 | 33750 | 687425 | 575 | 15800 | 3175 |
|  | **Waters** | 50 | 0 | 400 | 79375 | 0 | 75 |
|  | **Construction land** | 16950 | 75 | 5450 | 0 | 160800 | 225 |
|  | **Unused land** | 0 | 50 | 4925 | 100 | 300 | 90875 |
|  |  | **Change to 2090** | | | | | |
|  | **Area/(km^2^)** | **Cultivated land** | **Forests** | **Grassland** | **Waters** | **Construction land** | **Unused land** |
| **2070** | **Cultivated land** | 1222625 | 0 | 0 | 50 | 28750 | 0 |
|  | **Forests** | 0 | 1464100 | 22650 | 0 | 350 | 50 |
|  | **Grassland** | 0 | 44525 | 643450 | 925 | 17900 | 3875 |
|  | **Waters** | 0 | 0 | 500 | 79475 | 0 | 75 |
|  | **Construction land** | 22525 | 150 | 8425 | 0 | 169500 | 225 |
|  | **Unused land** | 0 | 75 | 5875 | 275 | 350 | 87825 |

**
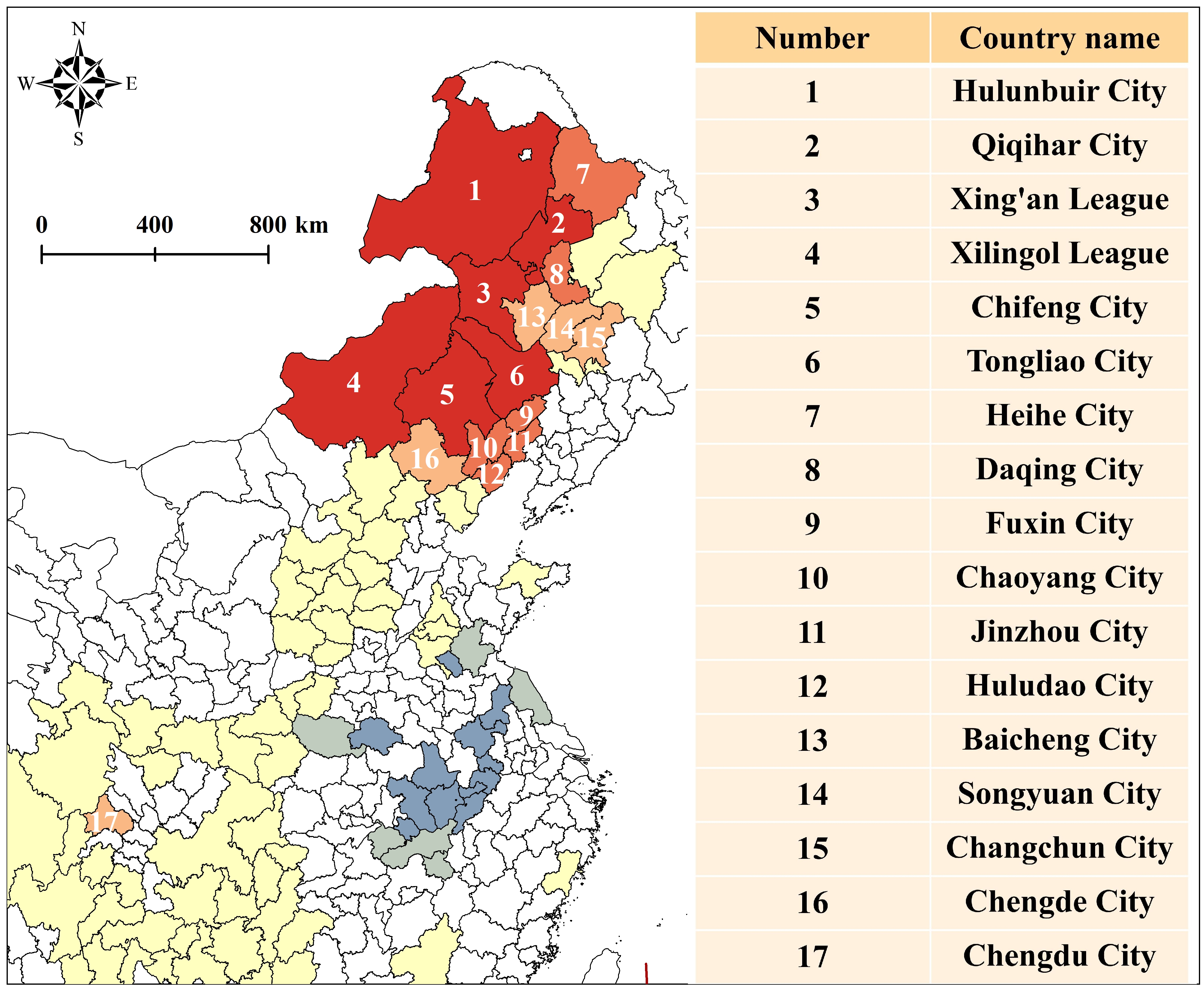
**

**Figure. S7**  Wild tending areas of *T. chinense*.
